# Multidimensional applications of prussian blue-based nanoparticles in cancer immunotherapy

**DOI:** 10.1186/s12951-025-03236-x

**Published:** 2025-03-03

**Authors:** Jiayi Zhang, Fang Wang, Zhaogang Sun, Jun Ye, Hongqian Chu

**Affiliations:** 1 Translational Medicine Center, Beijing Chest Hospital, Capital Medical University, Beijing, 101149 China; 2 Beijing Key Laboratory in Drug Resistant Tuberculosis Research, Beijing Tuberculosis and Thoracic Tumor Research Institute, Beijing, 101149 China; 3 State Key Laboratory of Bioactive Substance and Function of Natural Medicines, Institute of Materia Medica, Chinese Academy of Medical Sciences & Peking Union Medical College, Beijing, 100050 China

**Keywords:** Prussian blue, Nanoparticles, Photothermal therapy, Nanozyme, Ferroptosis, Cancer immunotherapy

## Abstract

Immunotherapy holds notable progress in the treatment of cancer. However, the clinical therapeutic effect remains a significant challenge due to immune-related side effects, poor immunogenicity, and immunosuppressive microenvironment. Nanoparticles have emerged as a revolutionary tool to surmount these obstacles and amplify the potency of immunotherapeutic agents. Prussian blue nanoparticles (PBNPs) exhibit multi-dimensional immune function in cancer immunotherapy, including acting as a nanocarrier to deliver immunotherapeutic agents, as a photothermal agent to improve the efficacy of immunotherapy through photothermal therapy, as a nanozyme to regulate tumor microenvironment, and as an iron donor to induce immune events related to ferroptosis and tumor-associated macrophages polarization. This review focuses on the advances and applications of PBNPs in cancer immunotherapy. First, the biomedical functions of PBNPs are introduced. Then, based on the immune function of PBNPs, we systematically reviewed the multidimensional application of PBNPs in cancer immunotherapy. Finally, the challenges and future developments of PBNPs-based cancer immunotherapy are highlighted.

**Graphical abstract:**

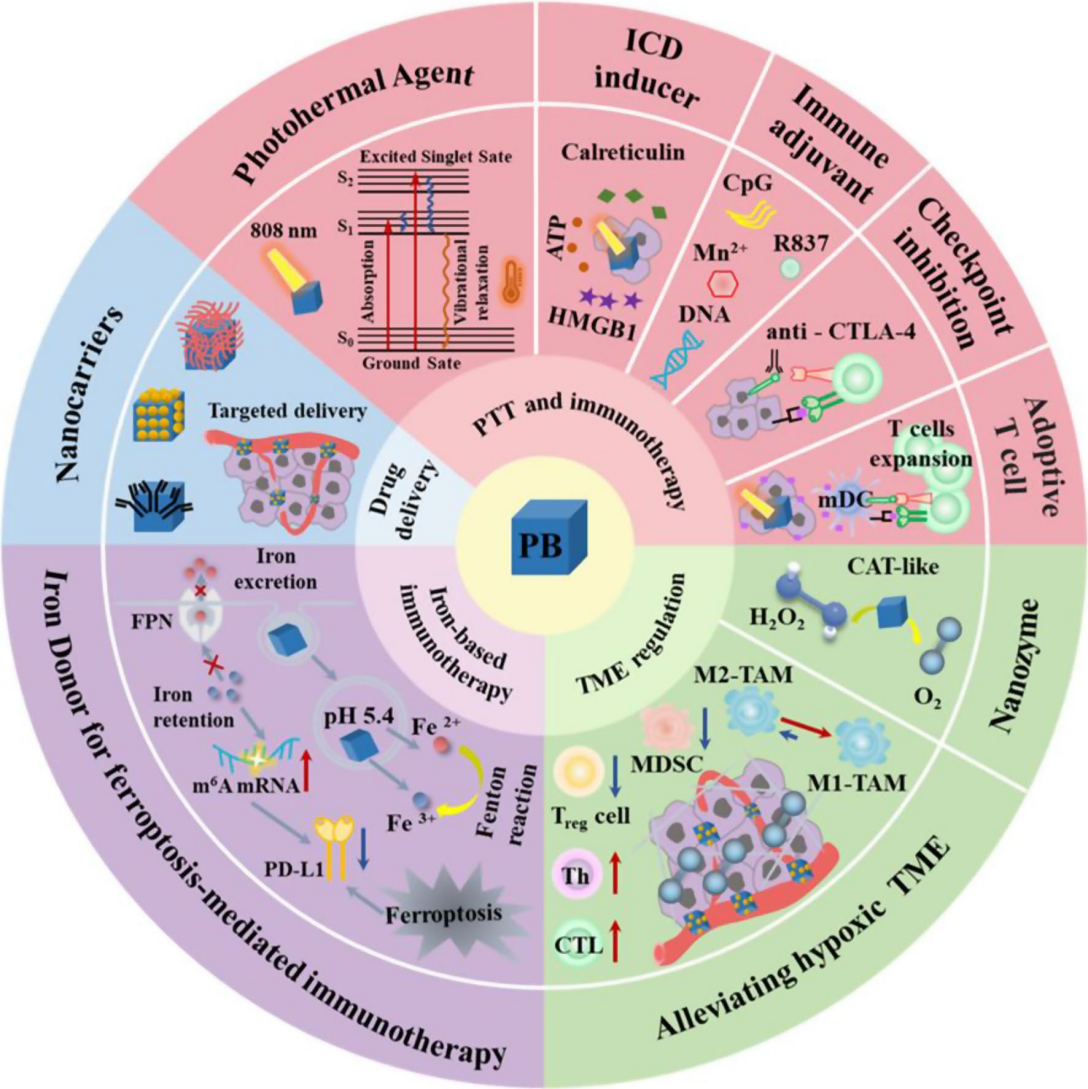

## Introduction

Cancer immunotherapy has emerged as a notable breakthrough in recent years. Compared with traditional approaches such as surgery, radiation therapy, and chemotherapy, immunotherapy utilizes the immune system of the host to attack and eliminate cancer cells. Additionally, immunotherapy can potentially induce immune memory, allowing the immune system to produce long-lasting protection against tumors, both locally and distantly, and reducing the risk of tumor recurrence [1, 2]. Following the revelation of immune response processes and mechanisms, various targeted cancer immunotherapies have been developed, such as immune checkpoint blockade (ICB) therapy, in which, the inhibitory signals of immune cells are targeted [3–5]; highly personalized adoptive cell therapy, in which, modified immune cells are transferred to the patient [6, 7]; and tumor vaccines that activate tumor-specific T cells and stimulate the tumor-specific antigen production [8]. Nevertheless, the personalization and uncertainty of antigens contribute to the differential response rates of immunotherapy [9]. In addition, hypoxic tumor microenvironment (TME) also induces the conversion of immune cells, such as tumor-associated macrophages (TAMs) are polarized into immunosuppressive M2 type, resulting in impaired antigen presentation and weak T-cell immune infiltration [10]. Moreover, immunotherapeutic agents often exhibit poor bioavailability, immunotoxicity, and decreased bioactivity [11]. Nanoparticles are promising tools for overcoming these challenges, either as immunotherapeutic agent carriers [12], immune stimulators [13], TME modulators, or in combination with existing immunotherapy strategies [14].

### The principles of tumor immunotherapy

The development of cancer is accompanied by a series of genetic mutations, which lead to the expression of neoantigens. Recent conceptual advancements over the past two decades have revealed that the immune system can both inhibit and facilitate tumor development and progression, a phenomenon known as cancer immunoediting [15, 16]. This process is characterized by three distinct stages: elimination, equilibrium, and escape. During the elimination phase, both the innate and adaptive immune systems collaborate to identify and eradicate transformed cells. Occasionally, tumor cells that survive the elimination phase move to the equilibrium phase, characterized by a restriction on net tumor growth, which can be sustained over time. Nevertheless, the persistent pressure exerted from adaptive immune system, in conjunction with the genetic instability inherent in tumor cells support tumor cells evade immune detection and destruction by selecting diminished immunogenicity.

The antitumor response mediated by both the innate and adaptive immune systems is the outcome of a series of sequential events called the “cancer-immunity cycle” [17]. the cancer-immunity cycle includes several key processes: (1) releasing: tumor-specific antigens (TSA) or tumor-associated antigens (TAA) generated through oncogenic processes are released; (2) presenting: neoantigens are captured by antigen-presenting cells (APCs) and then APCs process and present antigens on MHCI and MHCII molecules to lymphoid organs; (3) priming: naïve T cells located in lymphoid organs identify specific peptide-MHC complexes via their T cell receptors (TCRs), which facilitates the priming and activation of effector T-cells; (4) trafficking: the activated effector T cells exit the lymphoid organs and navigate through the bloodstream to survey peripheral tissues until they encounter their corresponding antigens within tumors; (5) infiltration: T cells infiltrate the tumor bed and migrate into TME, transforming into tumor-infiltrating lymphocytes (TILs); and ultimately, (6) attacking: T cells recognize and bind to cancer cells through the interaction between its T cell receptor (TCR) and peptide–MHC complex, and kill their target cancer cell. Immune attack results in the release of generate cell debris and antigens from dying tumor cells, which triggers the wider and deeper immune response in subsequent revolutions of the cycle. Nonetheless, the efficacy of the cancer-immunity cycle is compromised by tumor immune escape, which operates through four primary mechanisms: (i) evasion of immune recognition, (ii) suppression of antigen-presenting cell (APC) maturation, (iii) inhibition of T-cell infiltration within the tumor microenvironment (TME), and (iv) recruitment of immunosuppressive cells and molecules, accompanied by the upregulation of immune checkpoint molecules that directly inhibit T-cell activity.

Several immunotherapies based on the immune cycle have been developed: cancer vaccines, cytokines, immune checkpoint blockade (ICBs), immune cell-based adoptive cellular therapies, and tumor microenvironment regulation [18, 19]. however, each of these therapeutic interventions utilized in clinical practice have their own limitations. Cancer vaccines targeting specific tumor-associated antigens (TAA) or tumor-specific antigens (TSA) have made some progress in preventing or treating kind of tumors like Gardasil for uterine cancer and sipuleucel-T for advanced prostate cancer. However, most tumors have low antigenicity, co-delivery limitations of both antigen and adjuvant, high MHC heterogenicity, and limited targeting efficiency of antigens that hinder DC maturation, and finally, the inability to generate long-lasting T-cell immunity [20]. Although the efficacy of ICBs in many cancers is promising, several challenges persist. Firstly, ICBs demonstrate efficacy primarily in tumors highly immune infiltrated cancers referred to as “hot” tumors, whereas not in “cold” tumors that exhibit minimal immune cells infiltration. Secondly, cancer heterogeneity and individual differences contribute to inconsistent responses to ICBs [3–5]. Despite notable advancements in chimeric antigen receptor T-cell (CAR-T) therapy for hematological malignancies, its effectiveness in treating solid tumors remains limited, such as the lack of tumor-associated antigens, immunosuppressive TME and other problems that hinder the response of solid tumors to CAR-T therapy [6, 7].

### Role of nanoparticles in tumor immunotherapy

Due to their unique biological and chemical properties, nanomaterials provide new options and tools in addressing the limitations associated with cancer immunotherapy. Firstly, the adjustable size, structure, surface functionalization and other physicochemical properties of nanoparticles enable them to improve the delivery of immunotherapeutic agents. Nanomaterials can reduce off-target toxicity and immune-related adverse events associated with immunotherapeutics related to cytokines and monoclonal antibodies. Nanoparticles can induce immunogenic death (ICD) by co-delivering antigens and adjuvants to TME. Nanoparticles improve the unsatisfactory response rates of immune checkpoint blockers (ICBs) through promoting their accumulation in TME and remodeling of the TME to augment immunogenicity.

Among the inorganic nanoparticles, Prussian blue nanoparticles (PBNPs) have been widely studied in cancer immunotherapy. PBNPs are constituted by the bridge between the cyanide group (-C ≡ N-) and Fe(III)/Fe(II) and are one of the oldest metal-organic frameworks (MOFs) with a large specific surface area, high porosity, and abundant active sites [21, 22]. Therefore, PBNPs can act as nanocarriers for the targeted delivery and controlled release of immunotherapeutic agents. Furthermore, PBNPs possess multiple functions in cancer immunotherapy. First, PBNPs act as a photothermal agent (PTA) that converts optical energy into heat under near-infrared (NIR) absorption for tumor photothermal therapy (PTT). However, PTT based on PBNPs can only inhibit the growth of primary tumors and is ineffective in eliminating recurrent tumors and metastases. Excitingly, PTT has been shown to induce immunogenic death (ICD) of tumor cells, resulting in the release of tumor cell-associated antigens (TAAs) and damage-associated molecular patterns (DAMPs), providing an in situ “tumor vaccine”, thus it is a novel way to enhance immune stimulation when combined PBNPs-based PTT with immunotherapy [23]. Fernandes et al. modified PBNPs with CpG oligodeoxynucleotides, as a nanoimmunotherapy (CpG-PBNPs-PTT) to trigger strong antitumor immune responses with abscopal effects driven by T cell activation and long-term robust tumor-specific T cell memory by leveraging the photothermal heating characteristics of the PBNPs along with the immunostimulatory properties of both PBNPs-PTT and CpG [24]. In addition, PBNPs possess multienzyme-like activity including peroxidase (POD)-like activity that can inhibit hydroxyl radical (•OH) generation, catalase (CAT)-like activity that can transform H_2_O_2_ into O_2_, and superoxide dismutase (SOD)-like activity that can efficiently quench superoxide radicals (O_2_^•–^) [25]. Thanks to these properties, PBNPs hold great potential to protect cells against oxidative stress or alleviate tumor hypoxic microenvironment, which is also the key factor in inflammation control and TAMs polarization regulation [26–28]. Intracellular iron content is one of the conditions for ferroptosis. According to the chemical composition, PBNPs act as an exogenous iron donor (ID) to supply Fe^2+^/ Fe^3+^, thus they can catalyze H_2_O_2_ into highly toxic •OH by Fenton action to induce ferroptosis of tumor cells [29]. Importantly, there is a cross-talks between ferroptosis and anti-tumor immunity, involving cancer cells and immune cells within TME and ferroptosis can synergistically enhance the effects of immunotherapy [30, 31]. Therefore, PBNPs-mediated ferroptosis combined with immunotherapy may provide novel methodologies and insights for cancer therapy.

**Scheme 1:**
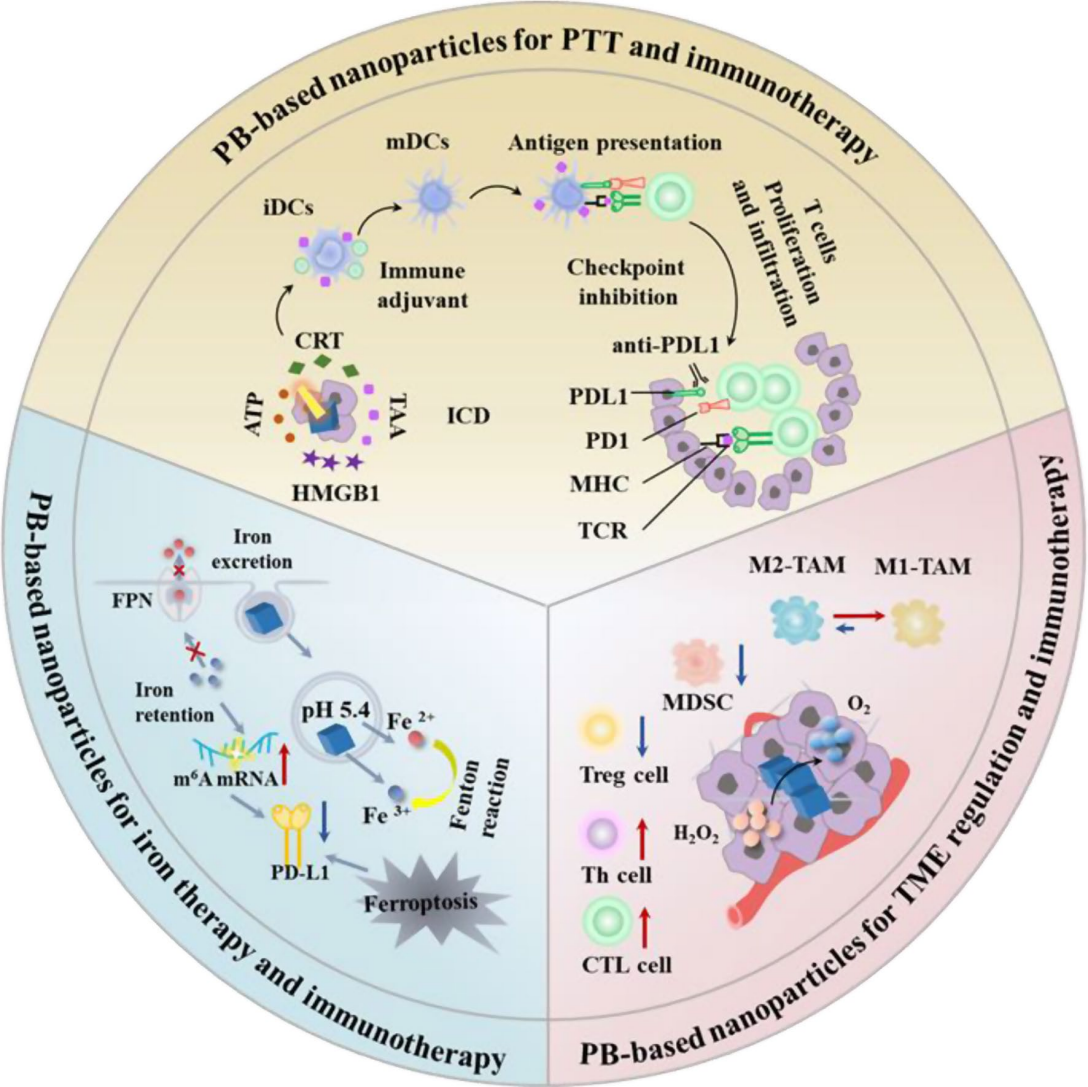
Multidimensional application of Prussian blue-based nanoparticles in cancer immunotherapy. ICD, immunogenic cell death; TAA, tumor-associated antigen; CRT, calreticulin; HMGB1, high-mobility group protein box 1; ATP, adenosine triphosphate; iDCs, immature DCs; mDCs, mature DCs; PD1, programmed death receptor 1; PDL1, programmed death receptor ligand1; MHC, major histocompatibility complex; TCR, T cell receptor; TME, tumor micro-environment; TAMs: tumor-associated macrophage M2-TAMs, M2 type tumor-associated macrophages; M1-TAMs, M1 type tumor-associated macrophages; MDSC, myeloid-derived suppressor cells; Treg cell, regulatory T cells; Th cell, helper T cell; CTL, cytotoxic T lymphocyte; m^6^A, N6-methyladenosine; mRNA, messenger RNA; FPN, ferroportin

Given their unique merits in composition, structure and properties, PB-based nanoparticles open a fresh door for the application of cancer immunotherapy. In this review, we first introduce the biomedical functions of PBNPs as a nanocarrier, a photothermal agent, a nanozyme, and an iron donor and the stability of PBNPs were discussed. Then, according to the different functions of PBNPs, highlight the recent progress of the multidimensional applications of PB-based nanoparticles in cancer immunotherapy (Scheme 1). These include (1) PB-based nanoparticles for PTT and immunotherapy, involving PTT-induced immunogenic cell death (ICD), PTT combined with immunoadjuvants, PTT combined with ICB therapy, PTT combined with adoptive T cell therapy; (2) PB-based nanoparticles for TME regulation and immunotherapy; (3) PB-based nanoparticles for iron therapy and immunotherapy and (4) PB-based nanoparticles for multimodal synergistic therapy involving immunotherapy. Finally, we outline the challenges faced by the PBNPs in clinical translation and discuss the future development trends in this field. This review will provide a paradigm for the study of PBNPs in the delivery of immune drugs and the treatment of immunoactive drugs, thereby realizing their great potential in anticancer.

## The biomedical functions of prussian blue nanoparticles

**Fig. 1:**
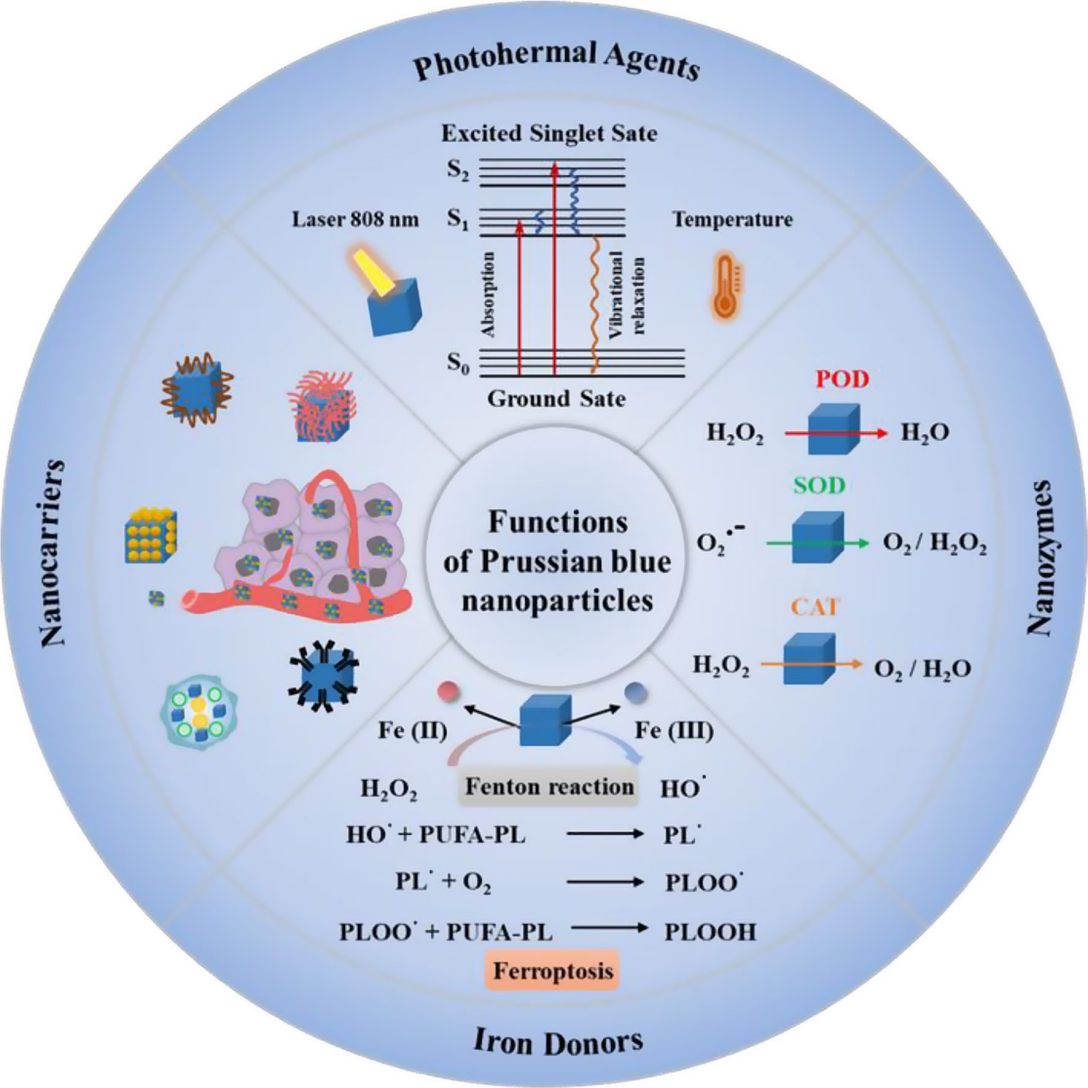
The main functions of Prussian blue nanoparticles. (1) the drug delivery function of PBNPs as nanocarriers; (2) the photothermal conversion function of PBNPs as photothermic agents; Reproduced with permission [56–59]. Copyright 2021, Royal Society of Chemistry. (3) the TME regulatory function of PBNPs as POD, SOD, and CAT-like nanozymes for oxygen generation; (4) the ferroptosis induction function of PBNPs as iron-donors via Fenton reaction. S_0_, the ground state; S_1_, the first electron-excited singlet state; S_2_, the second electron-excited singlet state; POD, peroxidase; SOD, superoxide dismutase; CAT, catalase; PUFA, polyunsaturated fatty acid; PL, phospholipid

Prussian blue was discovered in 1704 by the Berlin artist Diesbach, who produced a synthetic dye by simply mixing potassium ferrocyanide (K_4_Fe[CN]_6_) with ferric chloride (FeCl_3_) [32]. Subsequently, the molecular structure of PB was dissolved, including Fe(III) and nitrogen coordination, Fe(II) and carbon coordination to alternate arrangement in the cubic lattice site and to form a face-centred cubic cell with a dimension of 10.2 Å [33, 34]. The different formulas of PB can be obtained under different preparation conditions. When water molecules occupy vacancies in lattice defects, the molecular formula is Fe_4_^III^[Fe^I^(CN)_6_]_3_·nH_2_O, where the range of n is 14–16 and is commonly called ‘insoluble PB’, which is a larger crystal that easily aggregate and produce a precipitate [35, 36]. If the water molecule in the centres of the cubic cells is replaced by an alkali metal ion (monovalent cation), the molecular formula is AFe ^III^ [^II^(CN)_6_] ·nH_2_O, where n = 1–5, and is known as ‘soluble PB’, which is a lower dimensioned crystals that reach the size typical of the mesophase and exist as nanoparticles, forming clear deep-blue coloured colloidal solutions. In addition, numerous PB analogues (PBA) have also been discovered, with the general formula A_x_M_y_[M‘(CN_6_)]_z_, where A is a monovalent cation, M and M’ are bivalent and trivalent cations, respectively [37–39]. When the size is reduced to nanoscale, PB exhibits unique properties of nanoparticles, including adjustable structure and morphology, easy surface modification, and changes in optical and magnetic properties. Owing to their unique composition and structure, PBNPs exhibit a variety of excellent properties and functions in biomedicine. For example, the drug delivery function of PBNPs as nanocarriers, the photothermal conversion function of PBNPs as photothermic agents to ablate tumors, the TME regulatory function of PBNPs as nanozymes by generating oxygen, and the ferroptosis induction function of PBNPs as iron-donors via Fenton reaction (Fig. 1), which provides great convenience for constructing versatile PB-based nanoparticles for immunotherapy.

### The drug delivery function of prussian blue nanoparticles as nanocarriers

The potential of PBNPs as nanocarriers for drug delivery is linked to their advantages of definite and flexible structure, adjustable composition, and physical and chemical properties. The advantages and disadvantages of PB-based nanoparticles as nanocarriers compared with other inorganic nanocarriers will be discussed in terms of metal doping, pore size and surface modification.

#### Metal-doped prussian blue nanoparticles

The adjustable chemical composition allows PBNPs to introduce different metal ions or lanthanides by substituting or doping, thus obtaining Prussian blue analogues (PBA) or Prussian blue composite nanoparticles [40], retaining some of the original structure of PB while giving them new properties and functions. Transition metal elements, like Mn [41], Co [42], Cu [43], and Zn [44] have been used to replace Fe in PBNPs to optimize the performance of PB to a certain extent, such as the electron-hole effect, the enhancement of MRI contrast effect, or the enhancement of photothermal properties, providing powerful support for PBNPs in biomedical application. Shi et al. [45] prepared GPB NCs by doping Gd^3+^ into PB nanocrystals enables PB with tunable localized surface plasmon resonances (LSPRs) from 710 to 910 nm, which may be attributed to the position of Gd^3+^ in PB changing from a gap site to a lattice site as the amount of added H^+^ and Gd^3+^, resulting in high-enough free charge carrier (mainly [Fe (CN)_6_] vacancies) concentrations and electron transition changes in GPB NCs. Concurrently, they found that GPB NCs (~ 526 nm/RIU) have a higher plasma sensitivity than gold nanoshells (130 to 360 nm/RIU). Besides, Gd-doped hollow mesoporous PB (HGPB NCs) has higher photothermal conversion efficiency and stronger PA and MRI signals than HMPB NCs exposed to a laser. It is important that the improvement of these properties improves the efficacy of PB in tumor therapy and diagnosis. Manganese-doped Prussian blue nanoparticles (PBM) prepared by Wang group have lower redox potential (Mn^3+/2+^, 0.088 V) compared with PB (Fe^2+/3+^, 0.192 V) and charge transfer resistance (PBM is 2.98 Ω, PB is 4.83 Ω) [46]. As a result, PBM exhibits higher superoxide dismutase (SOD)-like activity, better GSH consumption and ·OH production than PB. More importantly, PBM realizes photothermal imaging-guided synergistic PTT and CDT, which not only initiates an ICD effect but also upregulate the STING pathway to activate the innate immune response of mice.

#### Tuning the morphology and pore size of prussian blue nanoparticles

Anti-tumor immunity is a cascade process, and its efficacy is controlled by many factors such as innate and acquired immune responses and tumor microenvironment. Therefore, PB-based nanoparticles are excellent nanocarriers for integrating traditional therapeutic drugs with immunotherapeutic drugs, providing an effective platform for combining multiple therapies with immunotherapy. The special Metal-organic frameworks (MOFs) structure with large specific surface area and high porosity permits PBNPs to capture not only monovalent cations (such as Cs, Tl) in the tetrahedral sites but also small molecules and complexes. Compared with other inorganic nanomaterials, PB can be used as a container of gas container such as CO, NO, H_2_, and can control the release of gas by photothermal heating for combined photothermal and gas therapy. Li et al. coordinated Fe(CO)_5_ on mesoporous PB by substituting -CN- with one of the CO groups of Fe(CO)_5_ [47]. The release amount of CO was controlled by irradiation time and intensity to avoid their binding to hemoglobin and thus reducing acute toxicity to normal tissues. However, compared to mesoporous (2 nm < pore size < 50 nm) and macroporous (pore size > 50 nm) nanoparticles, the pore size of PBNPs is less than 2 nm, which limits their loading of macromolecules [48]. Mesoporous silica nanoparticles (MSNs) with large pore sizes (10–30 nm) are effective delivery carriers for immune macromolecules (such as antigens, cytokines, antibodies) and vaccines, and draining into the lymph nodes. Thanks to the higher specific surface area and larger average pore size, hollow mesoporous Prussian blue nanoparticles (HMPBNPs) have been extensively explored to further improve the drug-loading capacity of PBNPs. HMPBNPs can be prepared by chemical etching of solid PB with strong acid or base, but the parameters of the hollow spheres, such as size and shell thickness, were almost uncontrolled [49]. In addition, it is difficult to achieve mass production of hollow PB nanoparticles through the above-mentioned synthesis strategy. Zhou et al. [50] prepared a series of hollow MnFe PBA nanospheres with adjustable nanostructures using sodium citrate (SC) as a structure regulator (represented by MnFe-PBA-SC). The hollow nanostructures and intrinsic cavities can carry a large amount of doxorubicin (DOX), showing a high loading efficiency of MnFe-PBA-SC_1.0_-FA (91.8%), which was higher than that in MnFe-PBA-SC_0.5_ (63.2%).

#### Surface modification of prussian blue nanoparticles

The presence of Fe^3+^ metal-centre unsaturated sites enable PBNPs to improve drug loading rate through coordination and electrostatic interaction and to achieve targeted drug delivery and controlled release easily through surface functionalization. For example, surface modification of the polyethylene glycol (PEG) improves the stability and dispersibility of PBNPs [51]. Hyaluronic acid (HA) surface functionalization promotes cellular uptake and prolongs the blood circulation half-life of PBNPs by targeting the overexpressed cluster of differentiation 44 (CD44) receptor of tumor cells [52]. DNA aptamers with specific recognition molecules have been used to functionalize PBNPs. Wu et al. demonstrated that DNA containing nucleolin aptamer (AS1411) and different base sequences conferred DNA-functionalized PBNPs targeting and higher enzyme activity than PBNPs alone [53]. To overcome the limitations of the blood-brain barrier impenetrability of NPs, Kim et al. used PBNPs-coated exosomes (Exo: PB) derived from the original cell line U-87 to achieve systemic targeted delivery [54].

The above examples demonstrate that PB-based nanoparticles can be synthesized and applied on demand by adjusting chemical composition, nanostructure, morphology and surface chemistry, which may be immune drug delivery nanocarriers with great translational clinical prospects.

### The photothermal conversion function of prussian blue nanoparticles as Photothermic agents

According to ligand field theory, the charge transfer (CT) between *ls* d^6^ t_2g_ Fe (II)-C and *hs* d^5^ t_2g_ Fe (III)-N produces the intense blue colour of PB and allows PB to absorb near-infrared (NIR) light energy (650–900 nm) [55]. PBNPs are regarded as a kind of promising PTA for tumor ablation due to their photothermal conversion function that converts absorbed NIR light energy into heat energy. The photothermal conversion process of PB can be illustrated by the Jablonski diagram (Fig. 1). Here, the light energy absorbed by the substance causes electrons in the ground state (S_0_) to transition to the high-energy first electron-excited singlet state (S_1_). These electrons in the S_1_ state are unstable and back to the S_0_ state through a non-radiative process known as vibrational relaxation, during which heat energy is released [56–59]. The molar extinction coefficient (ε) and photothermal conversion efficiency (η) are critical indices for assessing the photothermal treatment effect of PTAs. Yue et al. [60] used citric acid as a surface terminating agent to synthesize PBNPs with higher ε values (at 1.09 × 10^9^ M^− 1^·cm^− 1^) than carbon nanotubes (at 7.9 × 10^6^ M^− 1^·cm^− 1^) and Cu_2_ − xSe (at 7.7 × 10^7^ M^− 1^·cm^− 1^). Although the optical extinction coefficient of gold nanostructures in the NIR region is higher than that of PB, their photothermal conversion is highly dependent on their morphology and size, but their heat-induced melting and aggregation during PTT treatment seriously affect their PTT efficacy [61]. In addition, due to the high and unpredictable cost of gold, it is difficult to achieve clinical use. Graphene, as a representative of carbon-based materials, exhibits a high specific surface area, substantial drug loading capacity, significant light absorption capabilities, and elevated photothermal conversion efficiency. Nevertheless, their solubility, biocompatibility, and toxicity have been controversial [62, 63].

Compared with the commercial organic small molecule photothermal agent indocyanine green (ICG), Prussia blue nanoparticles have the following advantages as a photothermal agent: (1) PB has better monodispersity and stability in water than ICG, which is easy to aggregate in water [64]; (2) PB has better photothermal stability, the photothermal efficiency of ICG decrease after multiple light cycles due to photobleaching and thermal degradation [65], while that of Prussian blue nanoparticles remained almost unchanged; (3) The blood-circulation half-time of PBNPs is 2.78 h, which contribute to prolong the accumulation time of PBNPs at the tumor site and improve the efficacy of photothermal therapy [66]. ICG binds to plasma proteins nonspecifically in vivo and is quickly removed from the blood circulation, resulting in a plasma half-life of only 2–4 min [67].

However, due to the limited depth of penetration of the NIR (808 nm) structure, the η of PBNPs is only 20%. Wei et al. [68] fabricated PB-ytterbium (Yb) to raise electron density and orbital energy by doping the lanthanide metal element Yb into PBNPs, and the η increased up to 55.0% compared with that of PBNPs alone. PB is regarded as a P-type semiconductor, and enhanced photothermal effect has also been observed in platinum-doped PB [27]. With the increase of Pt content, the coordination between cyanogen and Pt leads to the decrease of [Fe(CN)_6_] vacancies, the maximum absorption peak is significantly redshifted, and the molar extinction coefficient increases at 808 and 980 nm. In addition, both the electronic structure calculation based on density functional theory (DFT) and the diffuse UV-vis spectroscopy measurements show that the introduction of Pt reduces the band gap, and the electron fraction density state indicates that the electronic circuit paths was increased to enhance non-radiative recombination for heat generation after Pt doping. The tissue penetration depth of NIR-I light still limited to the sub-centimeter scale, which makes PB-based nanoparticles more suitable for treatment of superficial tumors such as melanoma. Compared with the first NIR biological window (NIR I, 700 ~ 980 nm), the second NIR biological window (NIR II, 1 000 ~ 1 400 nm) can significantly reduce tissue self-heating and improve tissue penetration depth. Aggregation induced emission luminogens (AIEgens) represent an excellent candidate of versatile phototheranostic agents that can achieve the balance between radiative decay associated with fluorescence in the NIR-II and nonradiative dissipation involving reactive oxygen species (ROS)/heat to kill tumor cells [69]. Therefore, it is urgent to develop PB-based nanoparticles in the NIR II window.

### The tumor microenvironment regulatory function of prussian blue nanoparticles as Nanozymes

PBNPs function as a nanozyme, displaying a variety of enzyme-mimicking activities, including peroxidase (POD), catalase (CAT), and superoxide dismutase (SOD)-like activities [70]. Interestingly, the enzyme-like type and activity of PBNPs vary depending on the pH conditions. According to the Warburg effect, a differential pH gradient exists among tumor TME, and the normal blood circulatory system [71]. As shown in Fig. 1, with a value between 4.5 and 6.0 corresponding to the lysosome/endosome microenvironment, PBNPs exhibit POD-like activity and are capable of decomposing H_2_O_2_ into H_2_O. With a pH value between 6.5 and 6.8 corresponding to TME, PBNPs exhibit SOD-like activity and can effectively scavenge reactive oxygen species (ROS). With a pH value around 7.4 corresponding to a normal blood circulation microenvironment, PBNPs exhibit CAT-like activity and can reduce H_2_O_2_ to O_2_. Gao et al. reported that gadolinium (Gd)-doped PB enzyme coated in small hollow mesoporous silica NPs (HMSNs) produced HMSNs-PB-Gd with an excellent CAT-like activity, which considerably improved the hypoxic TME and further improved the photodynamic therapy (PDT) efficiency [72]. These activities enable PB-based nanoparticles to target tumor TME for maintaining redox homeostasis and relieving hypoxia. Notably, hypoxia and oxidative stress contribute to tumor immune escape; hence, PBNPs present great application potential in reversing TME immunosuppression.

### The ferroptosis induction function prussian blue nanoparticles as iron-donor

There are two different valence states of iron within the PBNPs, bivalent iron (Fe^2+^) and trivalent iron (Fe^3+^) [73]. Iron is an essential trace element in organisms, and its homeostasis considerably affects tumor progression [74]. Like other iron-based nanoparticles, PBNPs deliver exogenous iron to cancer cells by releasing Fe^2+^ and Fe^3+^, resulting in cellular iron overload and subsequent activation of ferroptosis (an iron-dependent regulatory form of cell death) [75, 76]. The Fe^2+^ catalyzes H_2_O_2_ to produce •OH via the Fenton reaction. Following this, •OH drives phospholipids (PLs) of polyunsaturated fatty acids to produce PL hydroperoxide, which is a characteristic of ferroptosis event (Fig. 1) [77, 78]. Nevertheless, iron leakage, H_2_O_2_ deficiency, and innate antioxidant system cause tumor cells to be insensitive to PBNPs-mediated ferroptosis. Therefore, the future design of PB nanosystems needs to consider the regulation of iron-related proteins, H_2_O_2_ supplementation, and the destruction of redox homeostasis to sensitized ferroptosis. Magnetite (Fe_3_O_4_), hematite (α-Fe_2_O_3_), magnetite (γ-Fe_2_O_3_) are the most common iron oxide nanoparticles (IONPs) used in biomedical applications [79]. Magnetic iron oxide nanoparticles (Fe_3_O_4_ or γ-Fe_2_O_3_) can effectively accumulate at the tumor site under the assistance of an external magnetic field. However, Fe_3_O_4_ is unstable and easily oxidized in air, which may lead to changes in properties such as magnetic and catalytic activity. Although γ-Fe_2_O_3_ nanoparticles are more stable, their catalytic activity is weakened by a reduction in Fe^2+^ content. Unlike iron-based nanoparticles, which rely on the input of exogenous iron to induce ferroptosis, ferritin is a major intracellular iron storage protein, and their autophagy degradation can trigger spontaneous ferroptosis in cancer cells by effectively releasing endogenous iron and enhancing intercellular iron levels [80]. Therefore, ferritin-based nanoparticles are a potential way to trigger endogenous ferroptosis. However, the application of ferritin-based nanoparticles is usually limited because of the yield and high cost compared with other materials [81]. The polymer coating of iron nanoparticles can influence immune-related responses, potentially either suppressing or enhancing immune activity. Branched polyethyleneimine-coated superparamagnetic iron oxide nanoparticles (SPIONs) have been shown to augment the Th1 polarization of human dendritic cells (DCs) [82]. In contrast, dextran-coated iron oxide nanoparticles (IONPs) exhibited immunosuppressive properties, as evidenced by a decrease in splenic lymphocyte proliferation and a reduction in the secretion of pro-inflammatory cytokines, including IL-1β, IL-4, IL-6, IL-10, and TNF-α [83]. Reportedly, there is a crosstalk between ferroptosis and anti-tumor immunity [84, 85]. A newly discovered mechanism of CD8 + T cell-mediated tumor killing promotes ferroptosis of tumor cells through the secretion of interferon (IFN)-γ [86]. Ferroptosis also promotes the transformation of TAMs to anti-tumor M1 phenotype [87]. The above theories provide a new direction for the construction of PBNPs with ferroptosis-inducing properties and immunotherapeutic functions.

### The stability of prussian blue-based nanoparticles

The application of PBNPs-mediated drug delivery, photothermal therapies, and photoacoustic imaging necessitate a series of processes involving circulation, extravasation, and targeted accumulation at specific sites, such as tumors. These processes require nanoparticles to maintain stability within the circulatory system for extended periods, potentially lasting several hours or even days. Therefore, it is essential to clearly define the conditions under which PBNPs exhibit stability at a physiological pH of 7.4 to ensure their safe and reliable utilization in medical therapies and diagnostics.

Pallavicini et al. prepared citrate stabilized PB nanoparticle (c-PBnp) and explored the effect of pH on PB stability [88]. They found that c-PBnp exhibit inherent stability and can be used both in vitro and in vivo studies at pH < 6, under which conditions they last for over 24 h. On the contrary, they rapid degradation at pH 7.4 accompanied by a reduction of the charge-transfer absorption of to 50% or less within a 24-hour period, and small pH changes from 7 to 8 significantly influence the decomposition rate of c-PBnp. According to the degradation mechanism of c-PBnp, due to the formation of hydroxyl complexes in the unstable Fe^3+^ center, the PB network is destroyed, resulting in the degradation of c-PBnp, dissolution, and disappearance from solution. Polymer-modified PBNPs protect them from degradation, yet that require 10 times the amount of PVP to wrap cubic PBnp.

After NPs is injected into the body, biomolecules present in biological fluids invariably create a recognized coating on the nanoparticle’s surface in less than 0.5 min, referred to as the “protein corona” by KA Dawson [89]. This phenomenon of surface “biotransformation” significantly modifies the pharmacological and toxicological properties of nanoparticles, as well as their potential therapeutic or diagnostic capabilities, often in unpredictable manners. As reported by Doveri group, PBNPs treated with HSA concentrations of 3.0 mg/mL or higher exhibited over 80% integrity of PBNP@HSA after 24 h in a buffer solution at pH 7.4 [90]. The quantitative results indicated that the presence of multilayer protein crowns on PBnp, the amount of HSA/PBnp (1500–2300) greatly exceeded the calculated value of single-layer HSA on PBnp (180 HSA/PBnp). At least 1500 HSA molecules are attached to a single cubic PBNP of 41 nm side. Consequently, the mean density of HSA molecules per unit area (nm²) is calculated to be 0.15 HSA/nm². The deprotonation of the H_2_O molecules bound to the surface Fe^3+^ ions of PBNPs with a pKa of 6.68, which are responsible for the blue-shift of PBNPs from 706 nm (acidic solution) to 685 nm (basic solution). The pH level affects the charge of the human serum albumin (HSA) corona, resulting in aggregation at approximately pH 5, where the zeta potential of PBNP@HSA is around zero, and this pH value corresponding to the isoelectric point of HSA. Pallavicini et al. also confirmed that PBNPs with a weak citrate coating exhibit enhanced stability due to the rapid formation of a protein corona in environments rich in proteins. c-PBnp did not influence cell viability on EA.hy926, NCI-H1299, and A549 cell lines across a broad range of concentrations [89].

Iron is integral to the process of ferroptosis. In the bloodstream, Fe^3+^ binds to transferrin, which facilitates its transport to cells via transferrin receptor 1. Once inside the cell, Fe^3+^ is reduced and released into the labile iron pool (LIP) within the cytoplasm, where excess iron is sequestered. The intracellular LIP predominantly exists in the form of Fe^2+^. Due to the inherent instability and high reactivity of Fe^2+^, it catalyzes the formation of hydroxyl radicals through the Fenton reaction. These radicals subsequently interact with polyunsaturated fatty acids (PUFAs) present in cellular and plasma membranes, leading to the generation of lipids peroxides. This cascade of events ultimately culminates ferroptosis [91]. What need to be explained is •OH production depends mainly on pH and Fe (II) ligand. For example, for Fe (II) hydrated ions, a Fenton reaction occurs at an acidic pH (e.g., pH < 3) to form •OH, while the Fenton-like reaction of the Fe (II) -citrate and Fe (II) -histidine complexes produce •OH when the pH of the solution increases from acidic to neutral. In addition, the oxidation state of iron is very important for its role in promoting ferroptosis: divalent iron, rather than trivalent iron, is involved in ferroptosis [92].

In conclusion, the application prospect of inorganic nanomaterials in cancer immunotherapy is not simply determined by their chemical composition, structural and morphological characteristics, biocompatibility and stability, as well as their intrinsic biomedical functions, but each has its advantages and disadvantages, so it is necessary to fully consider all the above factors, continuously explor the application potential of inorganic nanomaterials in cancer immunotherapy and overcom their own defects in order to be applied in clinical practice as soon as possible.

Although immunotherapy provides an anti-tumor therapy that regulates the body’s own immune system independently of the tumor. However, the occurrence and development of tumors are the result of changes in tumor ecological environment consists of tumor cells, stromal cells, immune cells, extracellular matrix and vascular system. The interaction between tumor cells and component promotes tumor invasion and metastasis by inhibiting anti-tumor immune response and inducing immune escape in tumor microenvironment. Therefore, it is necessary to develop combined treatment strategies targeting tumor and tumor microenvironment to achieve the inhibition of tumor growth, metastasis and recurrence in a more effective and personalized way.

## Prussian blue-based nanoparticles for cancer immunotherapy

With the revolution of the biomedical functions of PBNPs and the development of immunotherapies, the application of PB-based nanoparticles in cancer immunotherapy has been widely explored. In this section, PB-based nanoparticles for cancer immunotherapy are introduced and classified, including (1) PB-based nanoparticles for PTT and immunotherapy, (2) PB-based nanoparticles for TME modulation and immunotherapy, (3) PB-based nanoparticles for iron therapy and immunotherapy, (4) PB-based nanoparticles for multimodal synergistic therapy and immunotherapy. In addition, the different types of cancer immunotherapies based on PBNPs and their therapeutic mechanisms are summarized in Table 1.

**Table 1:** Multidimensional applications of PBNPs in cancer immunotherapy

Category	Materials	Mechanisms	Cells	Administration	Ref.
PBNPs for PTT and immunotherapy	PBNPs	PTT induces ICD	Neuro2a	Intratumoral injection	[104]
	CpG-PBNPs	Increasing antigenicity and immunogenicity by endogenous adjuvant synergies with exogenous adjuvant	Neuro2a	Intratumoral injection	[123]
	CpG-PBNPs	Triggering ICD and eliciting robust and persistent immunological memory by CpG-PBNPs-PTT	9464D	Intratumoral injection	[24]
	pPBNPs-CpG@TD	Augmenting ICD by photoactive nano immunomodulators	4T1	Intratumoral injection	[124]
	MnPB-MnO_x_	Activating the cGAS-STING pathway by PTT and ROS	4T1	Intravenous injection	[138]
	PBM	Mn(II) amplifies PBNPs-induced cGAS-STING	4T1, CT26	Intratumoral injection	[140]
	MiBaMc	Potentiating ICB by PTT and PDT	MC38, 4T1	Intravenous injection	[157]
	PB/PM/HRP/Apt	PTT and PD-L1 immunosuppression	4T1	Intravenous injection	[162]
	PBNPs	Reversing immunosuppression by inhibiting CTLA-4 and PTT	Neuro2a	Intratumoral injection	[163]
	αCD137-PBNPs	PTT, co-stimulating T cells by αCD137	SM1	Intratumoral injection	[166]
	MPB-3BP@CM NPs	PTT, inhibiting CD47 and reducing ATP and Lactate	HCT116	Intravenous injection	[171]
	PBNPs	PTT stimulating tumor-specific T cells expand ex vivo	U87, SNB19	Not specified	[183]
PBNPs for TME modulation and immunotherapy	HMPB/BLZ945/ Anti-SIRPα@ATRA @ fibrin	Relieving hypoxic TME and reprogramming TAMs to the anti-tumor M1 phenotype	HEPA1-6	Intratumoral injection	[193]
	LMWHA-MPB/HMME	Regulating macrophages converter, alleviating hypoxia, enhancing SDT	4T1	Intravenous injection	[194]
	G/APH-M	Enhancing radio-immunotherapy by modulating hypoxia and metabolism	GL261	Intravenous injection	[195]
	HPB-S-PP@LOx/siPD-L1	Modulating acidity combined with programmed death ligand-1 (PD-L1) siRNA (siPD-L1)	4T1	Intravenous injection	[199]
	SP94-PB-SF-Cy5.5 NPs	Remodeling TME by ameliorating hypoxia and PTT	HEPG2, HEPA1–6	Intravenous injection	[200]
PBNPs for iron therapy and immunotherapy	AuPB@LMHep	Enhancing immune response of anti-PD-L1 by ferroptosis and increasing m^6^A modification	Kasumi-1, C1498	Intravenous injection	[211]
	TK-M@Man-HMPB/HCQ	Facilitating TAMs polarization and mitigating hypoxia	4T1	Intravenous injection	[22]
PBNPs for multimodal synergistic therapy and immunotherapy	PMo@CCM	Enhancing immunotherapy via PTT/CDT and remodeling TME	4T1	Intravenous injection	[217]
	CS-1@PB[HM] NPs	Chemo-photothermal by inducing pyroptosis	MDA-MB-231	Intravenous injection	[218]
	M1-N@PBNPs	Boosting the theranostic performance of AIEgens using nano catalyzer, robust cancer immunotherapy	4T1	Intravenous injection	[219]
	M@P-PDR	PTT combined with DTX-enhance immunotherapy	4T1	Intravenous injection	[228]

CpG: Cytosine phosphoric acid Guanine; pPBNPs-CpG@TD, 1-tetradecanol (TD)-wrapped and CpG-loaded porous Prussian blue nanoparticles; MnPB-MnOx, MnOx was integrated onto the surface of Mn-doped PB nanoparticles; ROS: reactive oxygen species; PBM, Mn(III)-loaded Prussian blue; MiBaMc, microbial synthesis of Prussian blue MOFs decorated with bacteria membrane fragments and modified with mitochondria-targeting agent TPP and photosensitizer chlorin e6; PM, platelet membrane; HRP, horseradish peroxidase; Apt, aptamer; CTLA-4: cytotoxic T lymphocyte-associated antigen-4; αCD137, agonistic CD137 antibodies; MPB, microporous Prussian blue nanoparticles; 3BP, 3-bromopyruvate; CM, cell membrane; BLZ945, CSF-1R small molecule inhibitor; ATRA, all-trans retinoic acid; LMWHA, low molecular weight hyaluronic acid; HMME, ematoporphyrin monomethyl hether; G/APH-M, hollow Prussian blue loaded with Gboxin and Au-Pt nanozymes and coated with GL261 cell membrane; Lox, lactate oxidase; programmed death ligand-1siRNA, siPD-L1; SP94, HCC-specific targeting peptide SP94; SF, sorafenib; cyanine 5.5, Cy5.5; Hep, hepcidin; LM, leukemia cell-membrane; TK-M, hybrid membrane of macrophages and thylakoids; Man, mannose; HMPB, hollow mesoporous Prussian blue; HCQ, hydroxychloroquine; PMo, molybdenum (Mo)-doped Prussian blue nanoparticles; CCM, cancer cell membrane; CS-1, Cinobufagin; HM, hybrid membrane; M, M1 macrophage cell membrane; AIEgens, luminogens with aggregation-induced emission; M@P-PDR, tumor cell membranes-coated PLGA nanospheres encapsulated with Prussian blue, docetaxel (DTX) and imiquimod (R837); PDT: photodynamic therapy.

### Prussian blue-based nanoparticles for photothermal therapy and immunotherapy

#### Initiating immunogenic cell death by photothermal therapy

ICD is a unique form of cell death that differs from necrosis and apoptosis, and it plays a crucial role in triggering antigen-specific immune responses and generating immune memory [93]. ICD can be induced by specific stimuli, including viral infections [94], certain Food and Drug Administration (FDA)-approved drugs (such as Lurbinectedin) [95], and specific forms of radiotherapy [96] and phototherapy [97]. Dying tumor cells release antigens (antigenicity) and endogenous adjuvants (adjuvanticity), such as DAMPs that induce an “immune cold tumor” to transform into an “immune hot tumor,” eliciting a potent anti-tumor immune response [98, 99]. Furthermore, dying tumor cells, acting as TAAs, are engulfed by antigen-presenting cells (APC), undergo maturation, and then are processed and cross-presented to T cells, thereby promoting both innate and adaptive immunity in preclinical models [100]. PTT has traditionally been considered a local treatment, primarily due to the requirements of nanoparticle accumulation and laser coverage at the tumor site. Attractively, cancer ablation therapy based on PTT provides a novel way to induce the ICD of cancer cells, such as Au NPs, CuS NPs, and indocyanine green (ICG) have been used as ICD inducers [101–103]. PBNPs with tunable photothermal properties offer an opportunity for achieving optimal ICD, resulting in an “abscopal effect,” where the immune response extends beyond the treated tumor site.

**Fig. 2:**
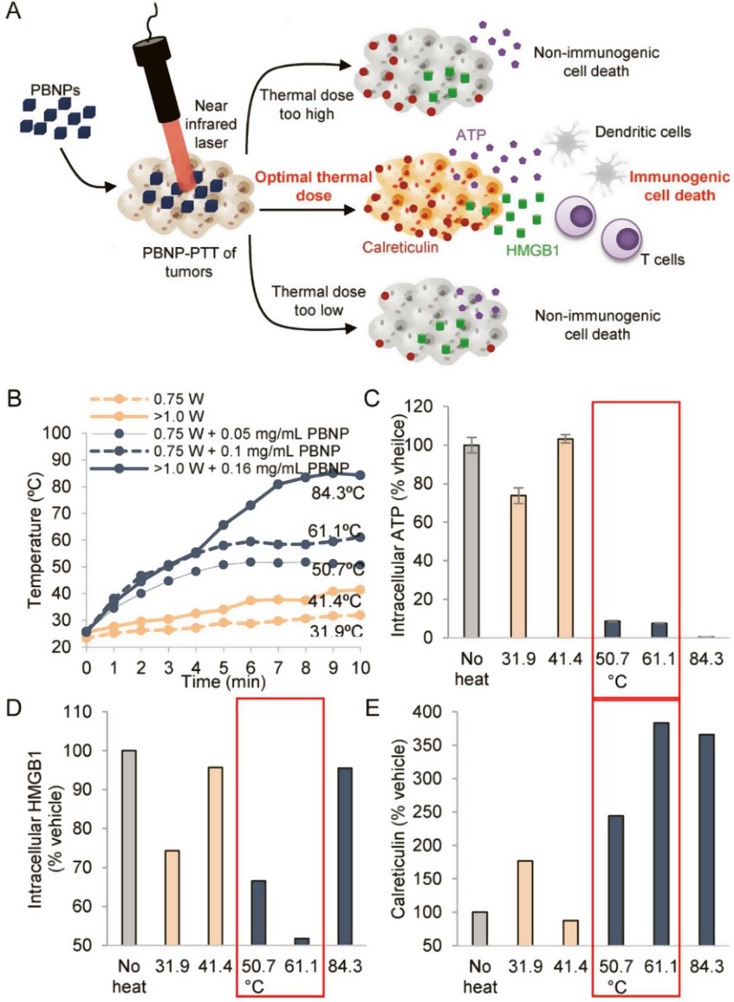
(**A**) Schematic illustration of Prussian blue nanoparticles-based photothermal therapy (PBNPs-PTT) generates a thermal window of immunogenic cell death. (**B**) Temperature–time profiles of samples containing ten million Neuro2a cells treated 0.75 W laser, > 1 W laser, 0.75 W laser + 0.05 mg·mL^− 1^ PBNPs, 0.75 W laser + 0.1 mg·mL^− 1^ PBNPs, and > 1 W laser + 0.16 mg·mL^− 1^ PBNPs. (**C**) Intracellular ATP, (**D**) Intracellular HMGB1 and (**E**) Surface calreticulin expression in the various treatment groups (as a % of the vehicle-treated group). Red boxes indicated the treatment temperature ranges for which all three markers of ICD are expressed/present (to varied degrees). Reproduced with permission [104]. Copyright 2018, Wiley-VCH-GmbH

Fernandes et al. utilized a neuroblastoma model with a lower neoantigen load to investigate the effect of PBNPs-PTT on inducing ICD (Fig. 2A) [104]. They discovered that varying thermal doses of PBNPs-PTT exerted different effects on the tumor cells in vitro and in vivo and the highest level of cell heating does not necessarily lead to enhanced ICD induction. Instead, they reported an optimal temperature range for effective ICD induction by PBNPs-PTT. In vivo, vaccine studies further confirmed that PBNPs-PTT at the optimal thermal dose, induced ICD and effectively eliminated Neuro2a cells, releasing endogenous adjuvant and promoting immune cell engagement, ultimately leading to the rejection of neuroblastoma tumor challenges. By employing a simple and widely applicable method to evaluate ICD, which involved measuring three biochemical correlates against the Cumulative Equivalent Minute thermal dose value at 43 °C (CEM43), they demonstrated that the thermal dose maximizing ICD correlates (5.6 log [CEM43]) provided the greatest protection against tumor and resulted in the highest long-term survival (Fig. 2B-E).

This is the first study on PBNPs-induced ICD by PTT that explored the optimum ICD effect of this photothermal nanoparticle under appropriate thermal dose (a function of temperature and reaction time). However, studies on PTT-induced ICD and standardized protocols for evaluating thermal doses of immune stimulation are still lacking. Therefore, future investigations need to focus on determining the temperature range, potentially offering universal photothermal immunomodulatory methods and expanding their application in cancer therapy.

#### Photothermal therapy combined with immunoadjuvants

Adjuvants were originally developed as vaccine components to enhance the immunogenicity and stability of the antigens [105]. The discovery of regulatory mechanisms underlying adjuvant activities offered great opportunities for their development. Adjuvants can be classified into immune agonists and antigen-delivery adjuvants per their mechanism of action [106]. Immune agonists are specific signaling molecules that trigger danger signals and promote the maturation and activation of APCs by targeting pattern recognition receptors (PRRs, such as toll-like receptors [TLRs]) [107, 108], which are the first line of defence in the natural immune response and facilitate the initiation enhancement of the adaptive immune responses [109]. Notably, different types of danger signals, such as pathogen-associated molecular patterns (PAMPs) [110] and DAMP [111], activate different PRRs. TLR agonists target the TLR pathway to enhance antigen presentation, costimulatory signal, and cytokine secretion [112, 113]. Another immunoadjuvant targets the cyclic guanosine monophosphate–adenosine monophosphate synthase (cGAS)–stimulator of IFN gene (STING) pathway, which leads to the secretion of type I IFN (IFN-I), thereby inducing APC maturation, upregulation of costimulatory signals, and enhancement of the ability to present or cross-present antigens [114]. Although adjuvants have presented excellent immune-stimulating effects, formidable challenges remain for the development of them in immunotherapy. Safety is the primary factor to be considered in developing adjuvants. Next, the mechanisms of action of many adjuvants are still unexplored, and the proposed mechanisms, including stimulating DCs and enhancing antigen uptake/cross-presentation, activating complement, and inducing cytokine/chemokine release, need to be further elucidated. Therefore, further in-depth research on adjuvants is warranted. Nanotechnology and materials science have promoted notable advances in immunoadjuvants [115]. In contrast to free adjuvants, the inherent nanosize effect enables PBNPs to act as immunoadjuvant that activates the cGAS-STING pathway through photothermal damage to the endogenous DNA of the tumor cells or as NC for delivering immunoadjuvants. The specific application of PBNPs combined with immunoadjuvants in tumor immunotherapy is discussed below.

CpG oligodeoxynucleotide (ODN) is a synthetic, single-stranded nucleotide containing unmethylated CpG motifs, which are recognized by TLR9 like pathogen-derived CpG DNA and activates downstream signal through the myeloid differentiation primary response protein 88 (MyD88)-dependent pathway to directly or indirectly innate immune responses [116, 117]. CpG ODN contributes to DC maturation and T-cell activation by activating TLR9 [118, 119]. However, susceptibility to nuclease degradation and poor cellular uptake because of electronegativity severely limits the therapeutic application of CpG ODN. Notably, PBNPs overcome the defects in CpG ODN delivery, along with improving the efficacy of anticancer immunotherapy in combination with CpG ODN and PTT [120–122].

**Fig. 3:**
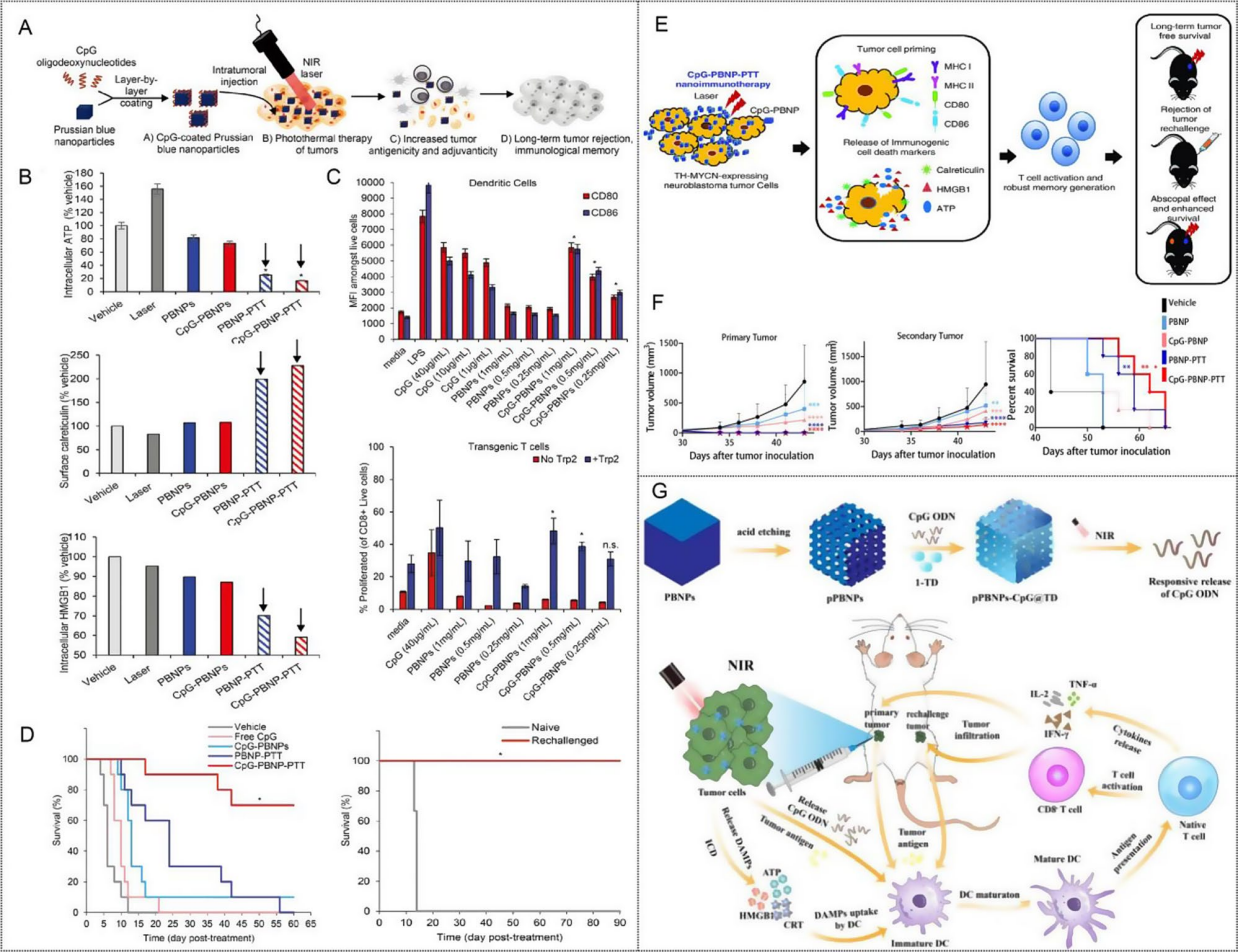
(**A**) Schematic illustration of CpG oligodeoxynucleotide-coated Prussian blue nanoparticle (CpG-PBNPs)-mediated nano immunotherapy for neuroblastoma. (**B**) Induction of immunogenic cell death by CpG-PBNP-PTT in vitro. (**C**) Immunostimulatory properties of CpG-PBNPs, including activation of dendritic cells and percentage proliferation of CD8 + T cells. (**D**) Effect of CpG-PBNP-based on long-term survival and rejection of tumor rechallenge. Reproduced with permission [123]. Copyright 2019, Royal Society of Chemistry. (**E**) Schematic of the mechanism of action of the CpG-PBNPs-PTT-based nanoimmunotherapy in the TH-MYCN model of NB. The CpG-PBNPs-PTT-based nanoimmunotherapy mediates tumor cell priming along with ICD administered at a specific thermal dose, leading to T cell activation and generation of potent T cell memory, which can elicit long-term, tumor-free survival, and rejection of tumor rechallenge in a TH-MYCN model of NB. (**F**) CpG-PBNP-PTT generates a potent abscopal effect, which induces complete tumor regression on the treated flank and significantly slows tumor progression on the untreated flank, and improving animal survival in the TH-MYCN NB model [24]. Copyright 2021, Wiley-VCH GmbH. (**G**) illustration of the construction of pPBNPs-CpG@TD for photothermal-responsive ICD-driven in situ anti-tumor vaccine-like immunotherapy [124]. Copyright 2023, Elsevier Ltd

In an example presented by the Rohan Fernandes group, CpG ODN was coated on PBNPs via a layer-by-layer scheme for CpG-PBNPs-mediated nano-immunotherapy (Fig. 3A) [123]. CpG-coated PBNPs possess photothermal eliminate tumor function under NIR laser irradiation after intratumoral injection. CpG-PBNPs-PTT induces ICD similar to that by PBNPs-PTT, through the release of endogenous adjuvants such as ATP, CRT, and HMGB1(Fig. 3B). By utilizing the inherent negative charge characteristics of PBNPs, PEI and CpG ODN are adsorbed to PBNPs layer by layer to synthesize CpG-PBNPs, which not only retains the PTT characteristics and antigenicity of PBNPs but also exerts the adjuvant properties of CpG ODN. In combination with the exogenous immunoadjuvant CpG, this increases both antigenicity and immunogenicity. DC co-culture with CpG-PBNPs and DC co-culture with free CpG were equally activated. DCs activated by CpG-PBNPs were co-cultured with CD8 + T cells in the presence of the in vitro model antigen Trp2, the proliferation of CD8 + T cell was increased than that activated with PBNPs (Fig. 3C). In the end, the increased antigenicity and adjuvanticity elicited by CpG-PBNPs-PTT results in complete tumor regression, long-term survival, and rejection of tumor rechallenge (Fig. 3D). This work provides examples of the delivery of other immune signals composed of tumor antigens, immune adjuvants, or other antigen-adjuvant combinations. However, the safety, potential toxicity, and long-term effects of these adjuvants in combination with PBNPs need to be further analyzed.

Many factors contribute to acquired immune tolerance in high-risk neuroblastoma (NB), including impaired T activity, reduced infiltration of immune cells, and deficiencies in antigen processing and presentation. T cells, as key immune cells for antigen-specific recognition, anti-tumor cytotoxicity and long-term antigen-specific memory, play an important role in tumor regression and sustained disease remission; however, impaired, or depleted T cell activity will not be able to effectively recognize or target tumor cells. Rohan Fernandes group [24] used clinically relevant TH-MYCN mouse neuroblastoma (NB) model (9464D) as the research object and used CpG functionalized stable PBNPs (CPG-PBNPs) to investigate the effect of CpG-PBNP-PTT-based nanoimmunotherapy (Fig. 3E). CpG-PBNPs-PTT “initiates” 9464D cells in vitro for recognition by immune effector cells to trigger a powerful systemic anti-tumor immune response and to trigger ICD as a function of thermal dose was preliminarily explored. This is also the first time that the thermal dose-dependent expression of costimulatory and antigen-presenting molecules has been studied. Subsequently, the efficacy of CpG-PBNPs-PTT in inducing complete tumor regression and robust long-term memory in a single tumor-bearing mouse in vivo was further demonstrated. Then, they co-cultured the splenocytes from any long-lived reattack mice with 9464D cells in vitro to confirm tumor-specific responses and immune memory produced by T cells. These in vitro studies involving T cells also represent a new dimension of PTT research. Finally, the abscopal effects generated by nanoimmunotherapy in a synchronous tumor model were evaluated (Fig. 3F). The above study demonstrates that the CpG-PBNPs-PTT nanoimmunotherapy drives potent systemic T cell responses in a syngeneic preclinical NB model and has the potential to achieve clinical transformation.

The in situ “tumor vaccine” effect generated by PBNPs-based PTT provides a paradigm for the development of novel therapeutic vaccines. However, the effectiveness of ICDs in TAA cross-presentation and T cell activation may be limited by inadequate innate immune stimulation in antigen-presenting cells (APCs) as well as the immunosuppressive character of TME, severely hindering T cell immune initiation. Therefore, the combination of ICDs with immune adjuvants that can directly activate APC and induce a co-stimulatory signal immune response initiated by T cells is expected to enhance anti-tumor effects. In Gu’s work, they developed a nanomodulator for photothermal ICD-driven in situ anti-tumor vaccine-like immunotherapy using porous PBNPs loaded with TLR9 agonist CpG and coated with 1-tetradecol (TD) (pPBNPs-CpG@TD) [124]. As shown in Fig. 3G, under NIR conditions, pPBNPs convert light energy into heat energy, and TD transforms into a liquid phase at the phase transition point (39 °C) to release CpG on demand and induce ICD synchronously with PTT, subsequently triggering a cancer immune cascade event. These include TAA release, TAA presentation, DC maturation, T cell initiation, and cytokine secretion to eradicate the primary tumor and prevent tumor recurrence.

When cGAS identifies the cytoplasmic double-stranded DNA fragment released by dead cells, tumor cells, or pathogens, cGAS catalyzes ATP and guanosine triphosphate to produce the second messenger 2’,3’-cyclic guanosine monophosphate, which binds and directly activates the STING protein [125]. Then, STING polymers recruit and activate TANK-binding kinase 1 (TBK1) which phosphorylates STING and interferon regulatory factor 3 (IRF3) and induces the production of IFN-Is and many other proinflammatory cytokines, regulating the natural anti-tumor-immune-responses in vivo [126–128]. Reportedly, Mn^2+^ can improve the sensitivity of the DNA sensor cGAS and its downstream adaptor protein STING [129] and act as a cGAS-STING agonist, directly inducing IFN-I and cytokine production in the absence of any infection, resulting in activation of innate immune response, and enhancing anti-tumor immunotherapy [130, 131]. Owing to its function, Mn^2+^ doping into nanoparticles for cGAS-STING-mediated tumor immunotherapy has garnered wide attention [132–136]. PB, as a classical coordination polymer, was designed with on-demand functional materials, which were referred to as PB analogues when the iron elements within were replaced by other transitional metal elements [37, 137].

**Fig. 4:**
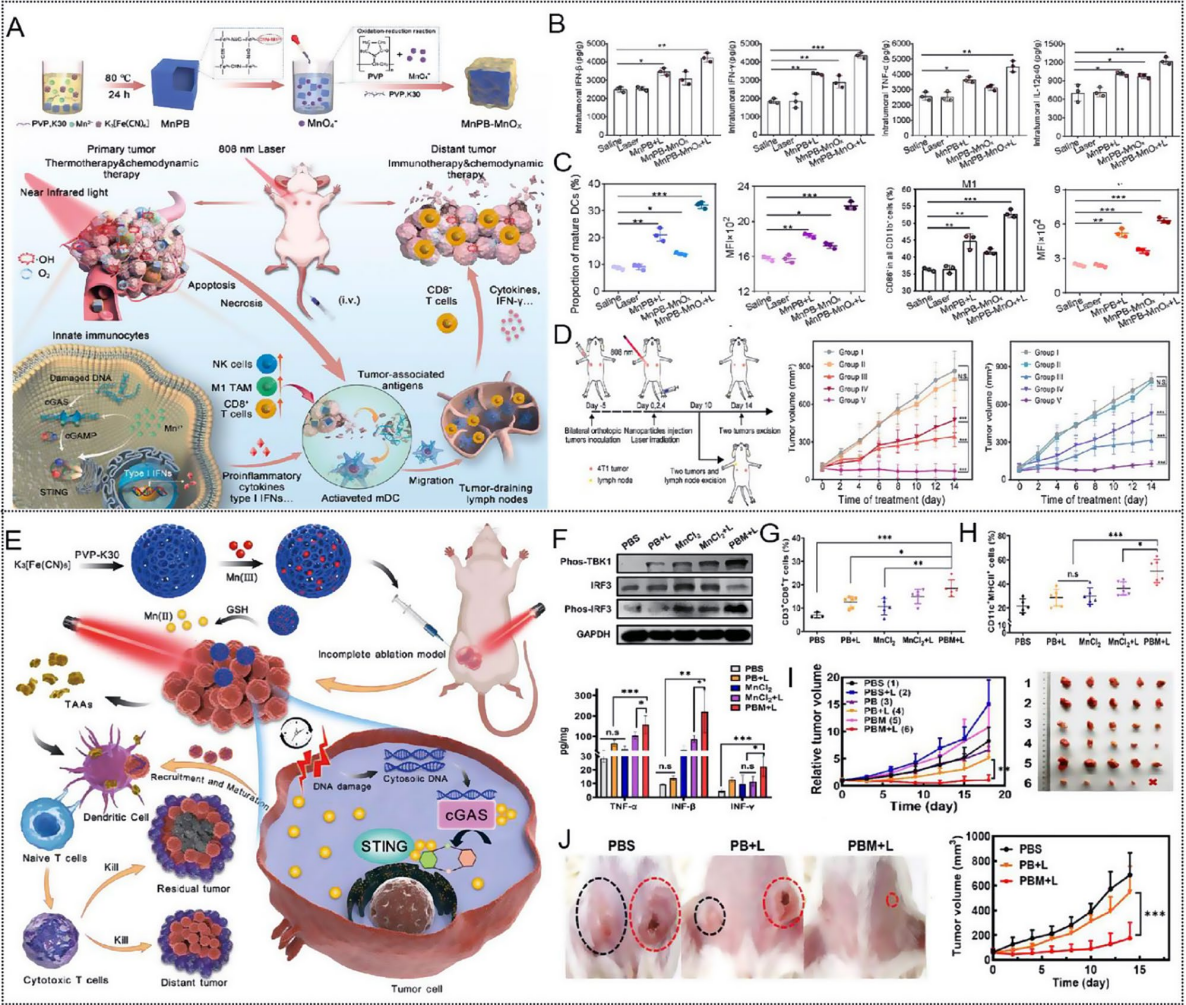
(**A**) Illustration of the construction of Mn-enriched MnPB-MnOx nanomedicines and the design principle of photothermal ablation synergizing with Mn^2+^-augmented cancer immunotherapy. (**B**) Proinflammatory cytokine type I interferons level in primary tumors from mice in each group on day 10 after various treatments. (**C**) DC maturation CD8, T cells, M1 (CD86 + macrophages in F4/80 + CD11b + CD45 + cells) and NK cells in primary. (**D**) Schematic illustration of the experiment design and time-dependent tumor volume curves for primary tumors (the former) and distant tumors (the latter) on mice after various treatments (group I: saline; group II: laser only; group III: MnPB + laser; group IV: MnPB-MnOx; group V: MnPB-MnOx + laser. The parameter of NIR-I laser was 808 nm wavelength, 1.5 W cm^2^, and 10 min exposure. Note: N.S. = not significant; **P < 0.01; ***P < 0.001. Reproduced with permission [138]. Copyright 2023, Elsevier Ltd. **E**) Schematic representation of the Mn(III)-doped nanoparticles for amplifying the cGAS-STING pathway, enhancing the antitumor immune response, and optimizing the efficiency of incomplete photothermal ablation therapy. **F**) The cGAS-STING activation in 4T1 tumor tissues by Western blot and the expression of TNF- α, IFN- β and IFN- γ in 4T1 tumor tissues by ELISA (*n* = 3). **G**) The production of cytotoxic T cells (*n* = 5) and **H**) dendritic cells (*n* = 6) in the tumor microenvironment. **I**) Tumor growth curves in the orthotopic breast tumor model (*n* = 5). **J**) Average tumor growth curves and representative photographic of the distant tumor. Data are shown as the mean values ± SD; Statistical significance was calculated by one-way ANOVA with Tukey’s test. ∗*p* < 0.05, ∗∗*p* < 0.01, ∗∗∗*p* < 0.001. n.s: no significance differences. Reproduced with permission [140]. Copyright 2023, Elsevier Ltd

A representative example is reported by the Wu group. First, Mn-doped PB (MnPB) NPs were synthesized by replacing Fe^3+^ with Mn^2+^, and then MnOx was bound onto the surface of MnPBNPs to construct an Mn-enriched photonic nanomedicines MnPB-MnOx for anti-triple-negative breast cancer (TNBC) PTT combined immunotherapy (Fig. 4A) [138]. The hyperthermy (45–50 °C) produced by MnPB under NIR light radiation combined with ROS generated by the Fenton-like catalyst of MnOx within MnPB-MnOx synergistically killed tumor cells and exposed numerous TAAs. In contrast, sufficient Mn^2+^ released from MnPB-MnOx instigated proinflammatory cytokine type I interferons production via up-regulating cGAS-STING pathway that enhanced DCs maturation and promoted the intratumoral recruitment of innate immune cells (including natural killer cells, M1-macrophages, and CD8 + T cells), resulting in efficient suppression of local/distant tumors growth while substantially preventing the mimic distant tumors (Fig. 4B-D). PTT-induced tumor ablation is generally accompanied by endogenous DNA damage in tumor cells, which activates the cGAS-STING pathway and initiates the downstream innate immune response [139]. Sun et al. proposed a strategy that synergistically enhanced immunotherapy utilizing dual immunoadjuvants (endogenous adjuvant combined with exogenous adjuvant) to address the limitation of incomplete photothermal ablation. They constructed a smart glutathione (GSH) responsive photothermal nanosystem Mn(III)-loaded Prussian blue (PBM) [140] by incorporating Mn element into PBNPs with hollow mesoporous and permeable lattice structure for optimizing photothermal ablation (Fig. 4E). Under laser irradiation, PBM treatment exhibited a hyperthermia ablation effect and induced numerous damaged cytosolic DNA to trigger the cGAS-STING pathway. Mn (II) is released from PBM after the interaction between Mn (III) and GSH, further amplifying STING activation (Fig. 4F). PBM + L treatment induced a robust immune response, enhancing DC maturation (Fig. 4G) and CTL production (Fig. 4H), thereby inhibiting both in situ residual and distant tumors (Fig. 4I and J).

Altogether, PBNPs can induce the release of TAAs and endogenous adjuvants (such as cytoplasmic DNA) while inducing PTT-mediated damage in tumor cells, indicating their potential as a new-generation nano-vaccine. Presently, the reported treatment strategy of PBNPs-PTT combined immune adjuvant synergistically enhanced immune stimulation, providing a reference for the design of a nano-adjuvant based on PBNPs-PTT. PBNPs may solve the application dilemma of more immune adjuvants.

#### Photothermal therapy combined with immune checkpoint blocking therapy

Immune checkpoints represent a class of biomolecules that exert specific immune resistance or suppression in the immune system. Notably, they are expressed on malignant tumors with epigenetic changes and on immune-related cells, such as programmed death ligand 1 (PD-L1) expressed on tumor cells and CTL-associated protein 4 (CTLA-4) and programmed cell death 1 (PD-1) expressed on T cells, which results in tumor immune escape, tumor progression, and metastasis [141–143]. The discovery of immune checkpoints led to monoclonal antibody-based ICB therapies, which reverse the immunosuppression by targeting immune checkpoints to reactivate the anti-tumor response of endogenous immune cells, particularly T cells [144, 145]. James P. Allison and Tasuku Honjo were awarded the Nobel Prize in Medicine Physiology in 2018 for their groundbreaking work. Notably, FDA-approved checkpoint inhibitors, namely anti-CTLA-4 (such as ipilimumab) antibodies and anti-PD-1 antibodies (such as nivolumab), have shown efficacy in metastatic melanoma, ovarian cancer, and non-small cell lung cancer [146–148]. However, the clinical response rates to these therapies are limited to specific patient subpopulations and resistance could be acquired during treatment [149, 150]. Low tumor immunogenicity and insufficient tumor-specific T cells (TILs, such as CD8 + T cells) infiltration in TME have been attributed to the low efficacy of checkpoint inhibitors [151, 152]. Nanoparticles with various functions have been utilized to overcome the limitations of ICBs, advancing cancer ICB therapy in a safer and more controlled manner [153, 154].

PB-based nanoparticles have shown the potential for delivering multiple types of immune checkpoint inhibitors to reduce their “on target but off tumor” effect, enhancing PTT-mediated tumor immunogenicity, increasing the number of T cells in lymphoid subsets, and promoting intratumoral infiltration of TILs by regulating immune microenvironment [155, 156]. Altogether, combining PBNPs with ICB may induce a systemic anti-tumor immune response, leading to the complete eradication of the primary tumor and potentially preventing tumor metastasis and recurrence.

**Fig. 5:**
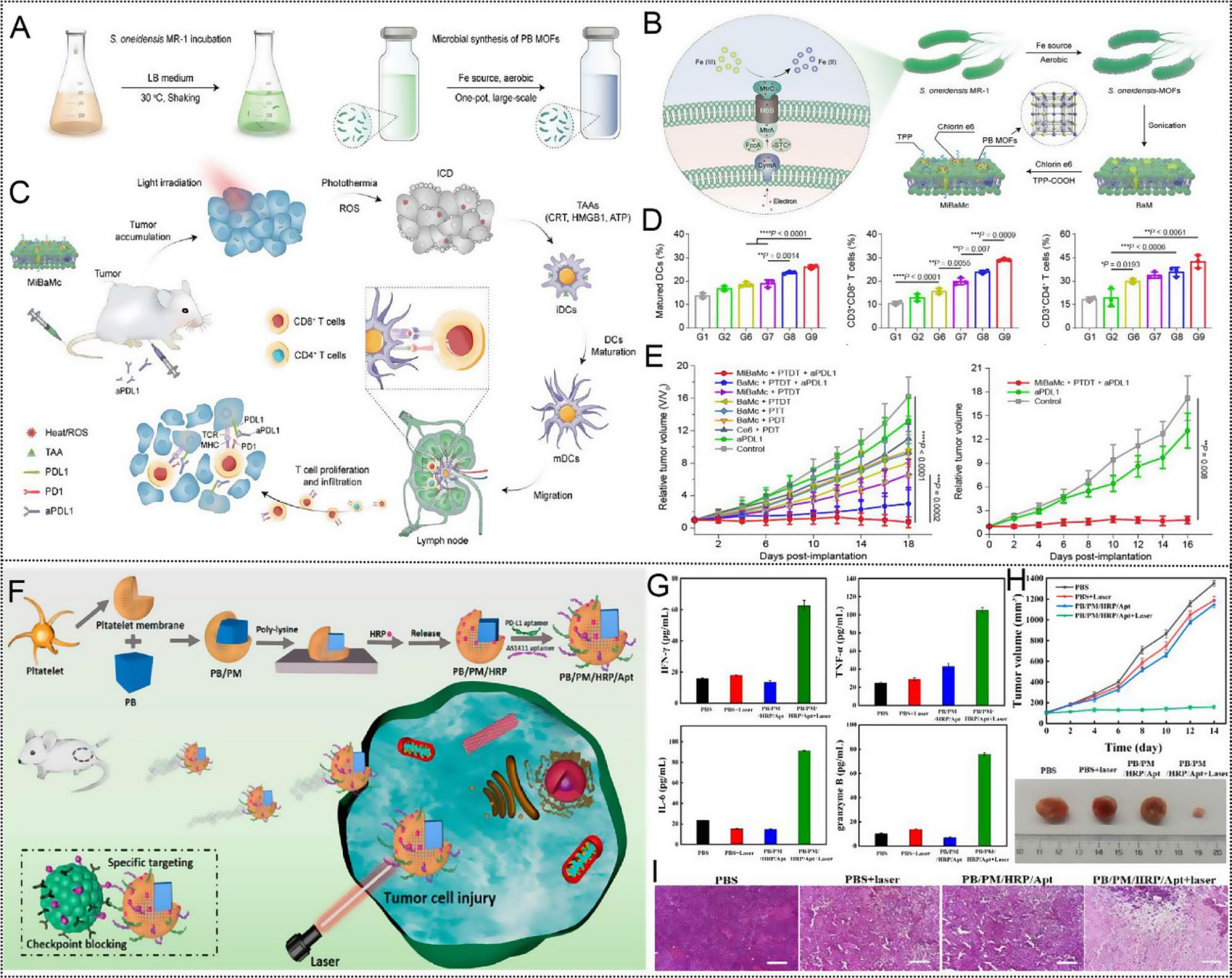
(**A**) Schematic illustration of the incubation of *S.oneidensis* MR-1 and subsequently one-pot large-scale microbial synthesis of FDA-approved Prussian blue MOFs. (**B**) Schematic illustration of the biological precipitation of PB MOFs coated *S. oneidensis* MR-1 hybrid (*S. oneidensis*-MOFs). (**C**) Schematic illustration of the mitochondria-targeting MiBaMc system-induced ICD combined with aPDL1 for enhanced tumor immunotherapy. (**D**) Quantitative analysis of the matured DCs, the CD3 + CD8 + cytotoxic Tcells and CD3 + CD4 + helper Tcells as a percentage of CD3 + lymphocytes based on flow cytometric results. (*n* = 3mice). (**E**) 4T1 (the former) and MC38 (the latter) tumor volumes of different groups were measured every 2 days (*n* = 5 mice). Statistical analysis was conducted by one way ANOVA with Tukey’s tests. n.s. represents none of significance, *P < 0.05, **P < 0.01, ***P < 0.001, ****P < 0.0001. Reproduced with permission [157]. Copyright 2023, Nature. (**F**) Schematic diagram of the synthesis and preparation process of PB/PM/HRP/Apt biomimetic nanocomposite and the mechanism of collaborative therapy. (**G**) The relative content changes of IFN-γ, TNF-α, IL-6 and granzyme B in tumor tissues. (**H**) The tumor growth curves of 4T1 solid tumor-bearing mice monitored every 2 days after different treatments (*n* = 5), and representative digital photographs of dissected tumors. (**I**) H&E staining images of 4T1 solid tumor sections obtained after injection of PBS, PBS + Laser, PB/PM/HRP/Apt and PB/PM/HRP/Apt + Laser. Scale bar: 100 μm. Reproduced with permission [162]. Copyright 2023, Frontiers

In a separate study, Zhao et al. developed a hybrid nanoplatform, MiBaMc, which combined a bacterial membrane with PB to enhance immune checkpoint blockade (ICB) immunotherapy on a large scale (Fig. 5A-C) [157]. MiBaMc was constructed using *Shewanella oneidensis* MR-1 bacteria-prepared PB metal-organic frameworks (*S. oneidensis*-MOFs)-decorated bacterial membrane fragments, further modified with ROS-generating chlorin e6 and mitochondria-targeting triphenylphosphine. MiBaMc specifically targeted mitochondria, inducing ICD in tumor cells under light irradiation via PTT and PDT. The released TAAs (such as CRT, HMGB1, and ATP) promoted DC maturation in tumor-draining lymph nodes and elicited T cell proliferation and infiltration. The highest percentage of DCs maturation, cytotoxic T lymphocytes (CTL) (CD3 + CD8 + T cells) and helper T cells (CD3 + CD4 + T cells) activation in the MiBaMc + PTDT + aPDL1 treatment group than other groups (Fig. 5D). As shown in Fig. 5E, MiBaMc-triggered PDT/PTT synergized with an anti-PD-L1 blocking antibody show a potent tumor inhibition in two tumor-bearing mouse models (immunogenic TNBC and colorectal cancer).

The aptamer is a structured oligonucleotide sequence (RNA or DNA) obtained by in vitro screening techniques, called exponential enrichment ligand phylogenetic evolution (Systematic evolution of ligands by exponential enrichment, SELEX) [158]. Compared with antibodies, aptamers have the advantages of a short screening cycle, small batch difference, simple amplification process, low cost, stable chemical structure, easy preservation and low immunogenicity and toxicity in production and application. What’s more, aptamers recognize target molecules through their three-dimensional conformation and interact with proteins with electrostatic attraction and hydrogen bonding, thus having higher specificity and affinity compared to antibodies [159, 160]. Therefore, aptamers are also known as “artificial antibodies”, and PD-L1/CTLA-4 aptamers have been used to replace antibodies and are widely used in cancer immunotherapy [161]. However, there are still many challenges in developing aptamers into tumor therapeutics. First, oligonucleotides are easily degraded by nucleases. Second, the aptamer is small in diameter, easy to be filtered by the kidney, and excreted quickly. Third, the biocompatibility and pharmacokinetic activity of aptamer in vivo are still unclear. As aptamer delivery tools, nanoparticles can improve tumor-targeting and accumulation of ICB antibodies and significantly alleviate the safety concerns of systemic antibody delivery, thereby improving therapeutic efficacy.

Guo et al. [162] synthesized a multifunctional therapeutic platform (PB/PM/HRP/Apt) with a unique working mechanism and good tumor therapeutic effect (Fig. 5F). Firstly, PBNPs were used as the nucleus to generate good photothermal conversion, and platelet membrane (PM) was coated to specifically target inflammatory sites and enhance PB accumulation in tumor sites, and then horseradish peroxidase (HRP) was modified on the surface to enhance the deep penetration of nanocomposites in cancer cells. In addition, PD-L1 aptamers and 4T1 cell aptamers AS1411 were mounted on nanocomposites to enable immunotherapy and enhance targeting. PTT combined with PD-L1aptamer can trigger cytokines release (Fig. 5G), including macrophage factor (IFN-γ and granzyme B) and proinflammatory factor (TNF-α and IL-6), resulting a strong immune response and tumor growth inhibition (Fig. 5H **and I**).

**Fig. 6:**
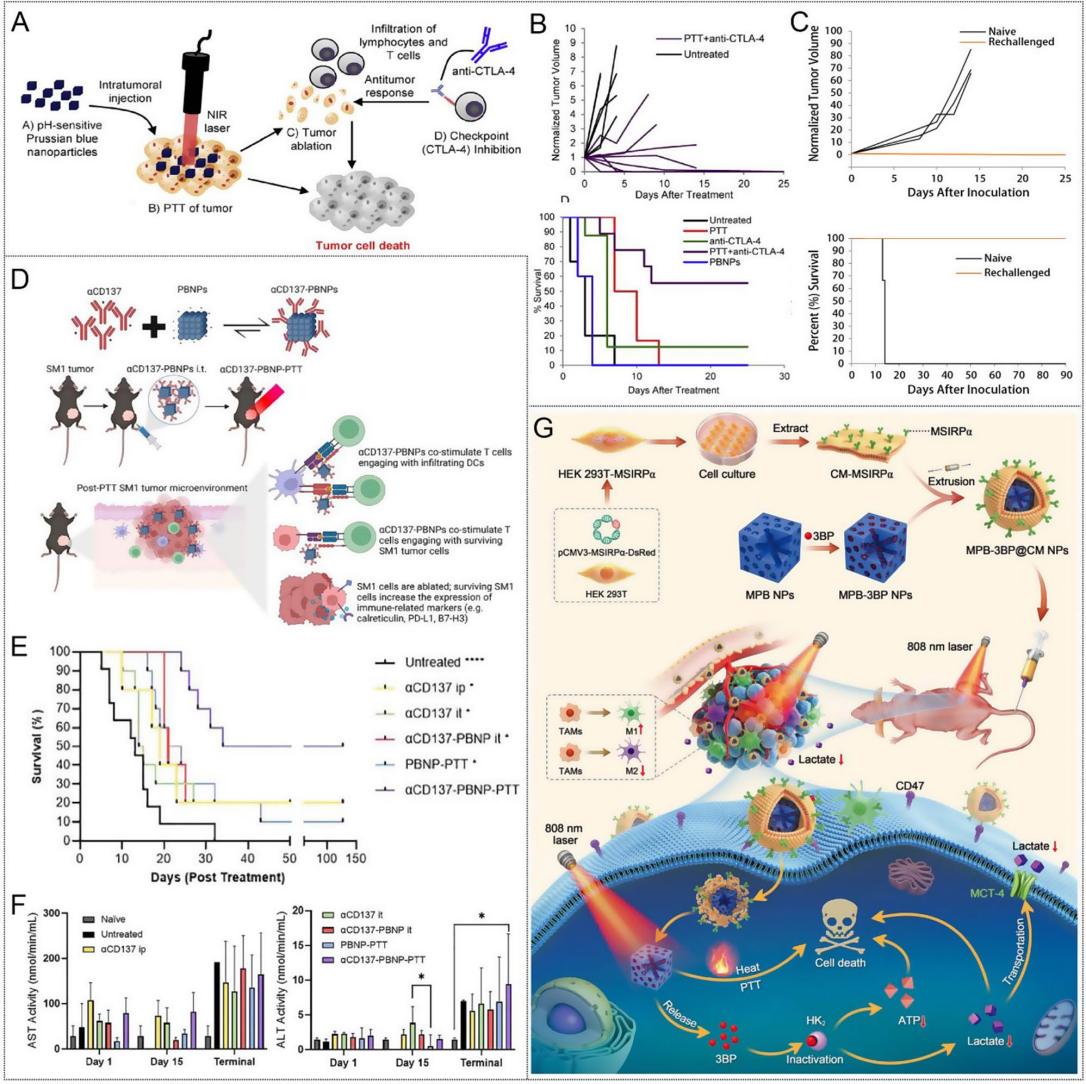
(**A**) Schematic illustration of PBNPs-based photothermal therapy combined with checkpoint (CTLA-4) inhibition for photothermal immunotherapy of neuroblastoma. (**B**) Normalized tumor growth curves and Kaplan-Meier survival plots of neuroblastoma mice receiving photothermal immunotherapy and the other groups (log-rank test; *p* < 0.05); (*n* ≥ 5/group). **X**) Tumor growth curves and higher long-term survival in the rechallenged group compared to naïve mice (log-rank test, *p* < 0.05); (*n* ≥ 3/group). Copyright 2017 [163], Elsevier Ltd. **D**) αCD137-PBNPs synthesis scheme, treatment regimen and proposed mechanism of action of αCD137-PBNPs-PTT to trigger anti-tumor immunity in SM1 melanoma. **E**) Kaplan-Meier survival curve of all mice in this experiment. **F**) Serum AST and ALT activity reveals no increased hepatoxicity in αCD137-PBNP-PTT-treated versus control and untreated SM1 tumor-bearing mice. * *p* < 0.05; ** *p* < 0.01; **** *p* < 0.0001 compared with αCD137-PBNP-PTT group. Copyright 2024 [166], Taylor & Francis. **G**) The construction of MPB-3BP@CM NPs and their implementation in combined therapy for CRC. Copyright 2024 [171], Nature

Another immune checkpoint-based photothermal immunotherapy was described by the Rohan Fernandes group. They combined PBNPs-based PTT with anti-CTLA-4 checkpoint immunotherapy to improve the outcome of neuroblastoma treatment in tumor-bearing mice (Fig. 6A) [163], presenting a notably higher rate of complete tumor remission and long-term survival (55.5%) compared with those of treatment with anti-CTLA-4 alone (12.5%) or PBNPs-PTT alone (0%) (Fig. 6B). This synergistic effect is attributed to PTT-induced immune response, which is complemented by anti-CTLA-4 therapy, reversing T-cell depletion and immunosuppression. Importantly, photothermal immunotherapy not only effectively eradicated tumors but also protected long-term surviving mice from tumor recurrence, in contrast to naïve mice that exhibited rapid tumor progression (Fig. 6C).

CD137 (4-1BB) is expressed on innate (DC and NK) and adaptive (T) immune cells as a member of the co-stimulating tumor necrosis factor (TNF) receptor superfamily (TNFRSF), which determines the degree of T cell activation, function, and survival, coupled with its unique ability to enhance anti-tumor and improve autoimmune responses, making it a promising target for clinical cancer immunotherapy [164]. Agonistic mAbs (αCD137) that mediate CD137 signaling have entered the clinic Agonistic mAbs; however, their safety in alone and in combination with therapy remains a concern due to systemic toxicity, which generally triggers acute liver inflammation. Therefore, there is a need to provide a strategy that reduces toxicity while maintaining efficacy [165]. Rohan Fernandes et al. [166] attached αCD137 electrostatically to PBNPs, forming αCD137-coupled PBNPs (αCD137-PBNPs). In vivo, αCD137-PBNPs were injected into SM1 mouse melanoma to colocated αCD137 and PBNP in the TME, thus achieving PTT and T cells co-stimulation, increasing immune-related markers expression on surviving SM1 cells surface. (Fig. 6D). This nanoplatform showed no hepatotoxicity and significantly improved survival (50%) of SM1 melanoma mice (Fig. 6E and F).

The interaction between cluster of differentiation 47 (CD47) protein overexpressed on numerous types of cancer cells and signal-regulatory protein α (SIRPα) receptor on myeloid cells to convey the “don’t eat me” signal, protecting cancer cells from macrophage-mediated phagocytosis that bears a superficial resemblance to the suppression of T cell activity by adaptive immune cell checkpoint PD-L1/PD-1 [167, 168]. Therefore, targeting innate checkpoint CD47-SIRRPα opens a new avenue for cancer immunotherapy. Using a recombinant protein composed of the extracellular region of CD47 or SIRRP-α to compete with the corresponding endogenous protein, affecting the normal binding of CD47 and SIRRP-α is an effective way to block the CD47-SIRRP-α interaction [169, 170]. Qian et al. [171] introduced a cell membrane biomimetic nanomedicine platform MPB-3BP@CM NPs for targeted combination therapy of colorectal cancer (Fig. 6G). Microporous Prussian blue nanoparticles (MPBNPs) serve as both photothermal sensitizers and drug carriers, loaded with 3-bromopyruvate (3BP) in their cavity and coated with genetically programmable cell membranes overexpressing MSIRPα (CM-MSIRPα) to synchronously target colorectal cancer (CRC) cells and enhance macrophage phagocytosis of CRC cells by competitively blocking the SIPα-CD47 interaction. 3BP (HK2), a hexokinase II inhibitor, reduces adenosine triphosphate (ATP) levels and lactic acid production by inhibiting glycolysis, which promotes the polarization of tumor-related macrophages (TAMs) toward anti-tumor M1 phenotypes. In addition, the photoacoustic imaging (PAI) capability of MPB-3BP@CM NPs allows precise mediation of PTT in vivo by MPB NPs, ensuring efficient tumor ablation.

Collectively, when combined ICB with PB-based PTT can enhance tumor immunogenicity while amplifying systemic immune response, thereby providing an immune-promoting microenvironment, avoiding T cell depletion, and regulating tumor growth and recurrence. The introduction of photothermal nanoparticles with immune triggering and regulatory functions into ICB required further in-depth study. The evaluation of biological activity and stability of nano-engineered immune checkpoint therapy systems have important implications for preclinical application.

#### Photothermal therapy combined with adoptive cell therapy

Adoptive cell therapy (ACT) has garnered considerable research interest in solid tumor treatment because of its successful application against haematological malignancies, such as anti-CD19 CAR-T cells showed high antitumor efficacy in patients with relapsed B-cell acute lymphoblastic leukaemia (B-ALL) and B-cell non-Hodgkin lymphoma, and the complete response rate was 70–94% in various trials. In CAR T-cell therapy, patient- or donor-derived T cells are genetically modified to express TAA-targeting CARs and generate an antigen-specific immune response in a manner independent of the presentation of major histocompatibility complex receptors [172–174]. However, CAR T-cell therapy has various challenges regarding treating solid tumors [175]. First, the non-specificity of antigenic targets, which are present in both tumor and normal tissues, can lead to severe toxicity [176]. Second, the complexity of TME (including abnormal vasculature and extracellular matrix) impedes the homing and penetration of CAR T cells. Additionally, continuously activated CAR T cells may cause adverse reactions associated with cytokine release syndrome. Recently, many efforts have been made to address these challenges, including improving T-cell expansion in vivo, overcoming the physical barriers and immune-suppressive environment to enhance T-cell penetration of solid tumors, and redirecting T-cell function through nanomaterial application in CAR T-cell therapy [177–180]. Other studies have explored the use of PTT-based NPs to realize endogenous T cell activation and targeted generation of CAR T cells in the tumor tissue [181, 182]. However, the heterogeneity of solid tumors poses a challenge for limited T cell populations to recognize the diverse tumor antigens.

**Fig. 7:**
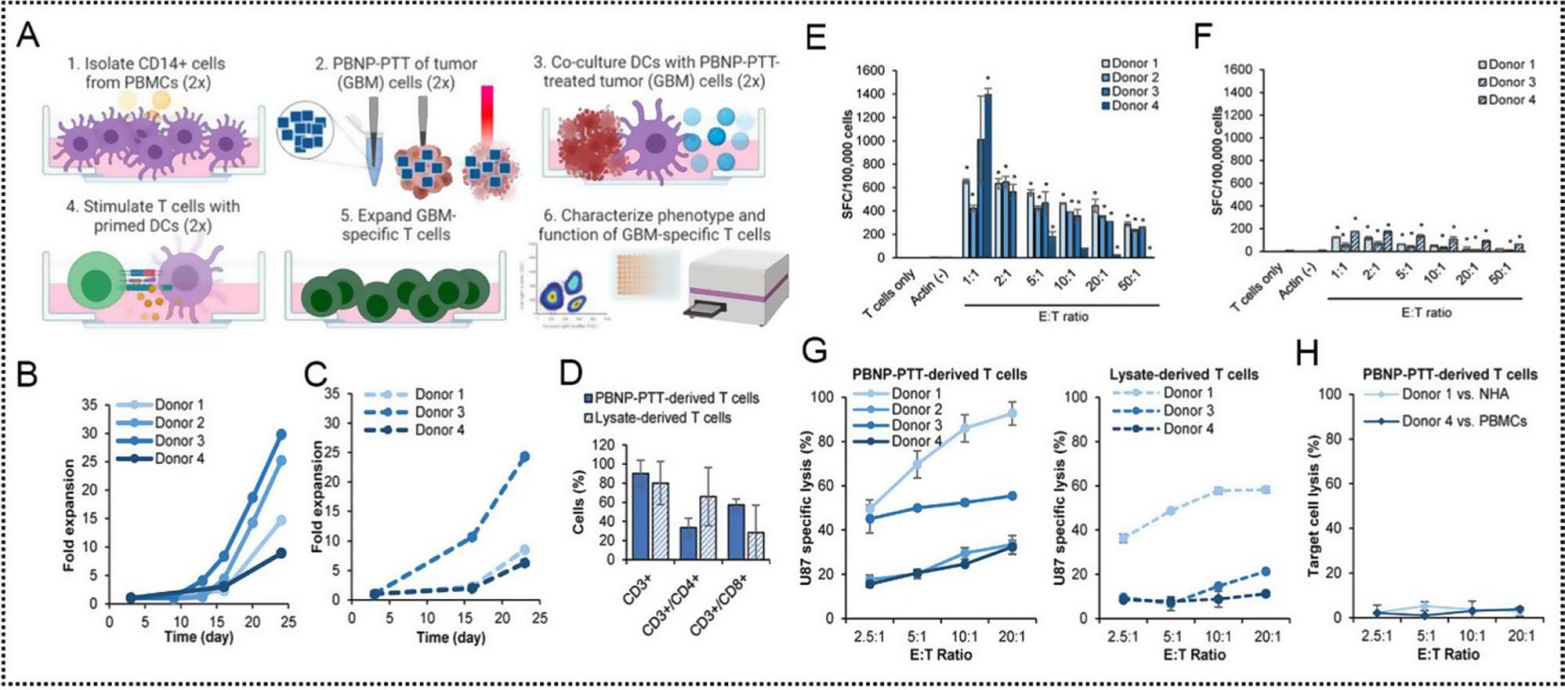
(**A**) Schematic illustration of the PBNPs-PTT-mediated tumor-specific T cell expansion scheme. (**B**) T cells developed by co-culturing with DCs primed with PBNPs-PTT-treated U87 cells and (**C**) U87 cell lysates (donor 2 is excluded due to availability of PBMCs) and their expansion. (**D**) Phenotype of T cell populations measured post-PBNPs-PTT-mediated ex vivo expansion. (**E**) T cells developed to target U87 cells via PBNPs-PTT-mediated and (**F**) U87 cell lysate-mediated expansion were co-cultured with U87 cells at the listed E: T ratios generated using a fixed number of T cells and decreasing number of target cells, and IFN-ɣ release (*n* = 2/group). *P < 0.05 versus actin. (**G**) U87-specific T cells developed via PBNPs-PTT and lysis were co-cultured with U87 cells at the listed E: T ratios for 4 h. Cytotoxicity was measured by calcein release (*n* = 2 replicates/donor). (**H**) U87-specific T cells developed via PBNPs-PTT (donor 1 or donor 4) were co-cultured with NHAs (donor 1) or PBMCs from the corresponding healthy donors (donor 4) at the listed E: T ratios. Cytotoxicity was measured by calcein release. Values represent mean ± standard deviation (*n* = 2/group). Reproduced with permission [183]. Copyright 2023, Elsevier Ltd

To address the challenge of targeting a wide array of antigens in solid tumors, Fernandes et al. demonstrated the potential of PBNPs-PTT-mediated immune stimulation for expanding tumor-specific T cells ex vivo [183]. This approach underscores the potential of ACT in the clinical treatment of patients with glioblastoma. Specifically, DCs were pretreated in a medium containing IL-4 and granulocyte-macrophage colony-stimulating factor (CSF) with CD14 + cells isolated from peripheral blood mononuclear cells (PBMCs) of healthy donors. Subsequently, PBNPs-PTT-treated tumor cells (GBM cells) were co-cultured with these DCs to produce a diverse and effective population of GBM-specific T cells for ATCT (Fig. 7A). Unlike direct lysis or heating of tumor cells, the use of PBNPs-PTT for heating can increase the cytotoxicity and immunogenic response of tumor cells in a thermal dose-dependent manner. Compared with T cells developed using U87 cell lysates that expanded only 6–24 folds, PBNP-PTT-treated U87 cells resulted in 9–30 folds of T cell expansion, wherein 90.1% of CD3 + T cells expanded, with 33.5% being CD4 + and 57.3% being CD8 + T cells (Fig. 7B-D). Furthermore, PBNPs-PTT-treated T cell products exhibited enhanced targeting of U87 cells (up to 647 times higher than control cells) and increased IFN-γ secretion in a dose-dependent manner (E: T ratio) compared with those expanded using U87 cell lysates (Fig. 7E **and F**). Importantly, T cells amplified via PBNPs-PTT exhibited higher specificity to target U87 cells and were more effective in killing cancer cells, but they did not affect normal human astrocytes and cytotoxicity of PBMCs from the same healthy donor because of the lack of autoreactivity (Fig. 7G). Similarly, the E: T ratio showed that the number of targeted U87 cells eliminated by T cells generated through U87 lysis was 2- to 3-fold lower than those developed using PBNPs-PTT (Fig. 7H). Furthermore, the PBNPs-PTT-mediated T cell expansion platform proved versatile and universally applicable, as evidenced by its effectiveness with another GBM cell line, SNB19 cells.

Although PBNPs-PTT has successfully stimulated and expanded tumor-specific T cells in vitro, this represents the first proof of concept that immunostimulatory photothermal NPs could serve as an ATCT for patients with solid tumors. However, isolated T cells may not persist or be depleted after infusion; therefore, future exploration should focus on the breakthrough of PB nanoplatform in the delivery of CAR transgenes and in vivo expansion of T cells for broader implementation of photothermal nanoparticles in immunotherapy. In summary, greater anticancer effects have been attained by versatile PBNPs to build communication between immunotherapy and other therapies than a single approach. Therefore, future work should focus more on vetting the collaborative mechanisms by which PBNPs synergistically with current tumor therapies to broaden their applications in tumor immunotherapy.

### Prussian blue-based nanoparticles for tumor microenvironment modulation and immunotherapy

To sustain malignant growth, the tumor stimulates angiogenesis [184]. However, abnormal structure and function of vasculature in turn lead to tumor microenvironment hypoxia [185]. Thus, the oxygen levels in the TME decline by < 10 mmHg, compared with 40–60 mmHg in the normal tissue [186]. Hypoxia leads to the establishment of immunosuppressive TME, which is an important limiting factor of immunotherapy and a prime cause of treatment failure [187]. For example, hypoxic TME promotes tumor cells to secrete several macrophage CSF (MCSF), which binds to the CSF-1 receptor (CSF-1R) expressed on macrophages to recruit and stimulate the polarization of M2 macrophages [188]. M2 macrophages increase the infiltration of regulatory T (Treg) cells and induce PD-L1 expression via releasing the anti-inflammatory factor (such as IL-10), which is the main cause of supporting postoperative tumor recurrence, whereas anti-tumoral M1 macrophages exhibit immune activation by promoting antigen presentation [189]. Therefore, alleviating hypoxic TME provides a strategy to reprogram the immune TME via TAMs polarization from the M2- to M1- phenotype.

CAT-like nanozymes, a promising hypoxia-modulatory nanomaterial, generate endogenous oxygen and present the advantages of high stability, low cost, and controllable and adjustable enzyme activities [190]. PBNPs internal electron transfer occurs between high spin Fe^3+/2+^ ions and low spin (CN)_6_^3-/4-^ ions allowing it to be used as a CAT mimics enzyme to convert H_2_O_2_ (concentration range from 500 to 1000 µM) into O_2_ in TME [191]. Consequently, PBNPs-assisted alleviation of TME hypoxia has been proven in cancer immunotherapy [43, 192].

**Fig. 8:**
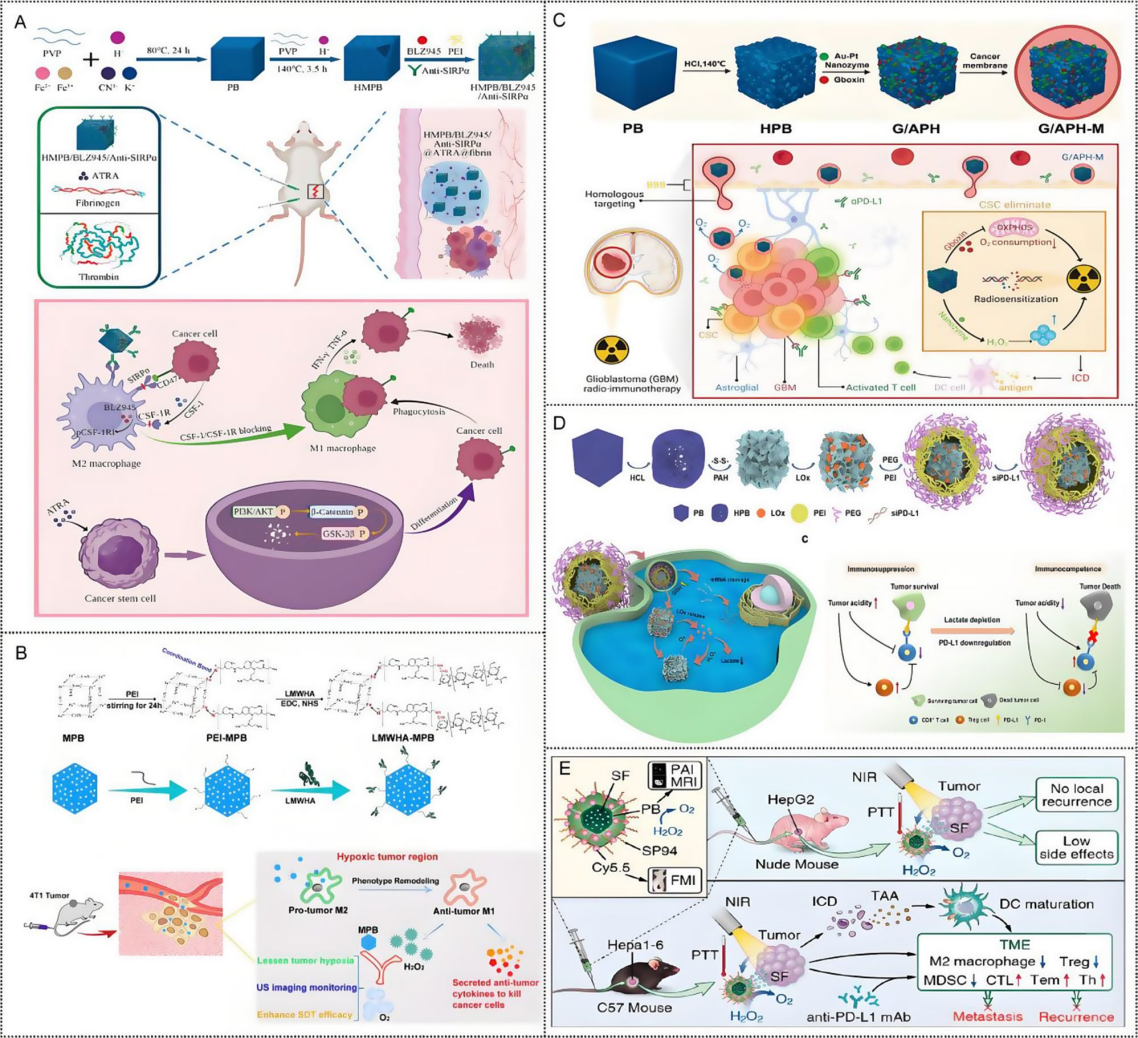
(**A**) Schematic illustration of the preparation process and behavior of HMPB/BLZ945/anti-SIRPα@ATRA@fibrin for cancer immunotherapy in vivo. The immunotherapeutic gel at the surgical site released ATRA and HMPB/BLZ945/anti-SIRPα to induce CSC differentiation and TAMs reprogramming. Reproduced with permission [193]. Copyright 2023, Elsevier Ltd. (**B**) Schematic illustration of the synthetic of LMWHA-MPB nanoparticles and HMME-loading LMWHA-MPB nanoparticles inhibits the proliferation and metastasis of 4T1 tumor in vivo [194]. Copyright 2019, Europe PMC. (**C**) Schematic illustration of the preparation of G/APH-M and G/APH-M-based radiotherapy in combination with anti-PD-L1 to enhance glioblastoma radio-immunotherapy [195]. Copyright 2023, Wiley-VCH GmbH. (**D**) Schematic illustration of lactate consumption combined with siPD-L1 to synergistically improve tumor immunotherapy [199]. Copyright 2023, Elsevier Ltd. (**E**) Schematic illustration of the multifunctional nanoplatform (SP94-PB-SF-Cy5.5 NPs) for HCC-targeted multimodality imaging and combined PTT/SF treatment. Reproduced with permission [200]. Copyright 2019, American Chemical Society

#### Reprogramming tumor-associated macrophages

As an example, Huang et al. [193] constructed an immunotherapeutic in-situ fibrin gel (HMPB/BLZ945/anti-SIRPα@ATRA@fibrin) by co-encapsulating anti-signal regulatory protein (SIRP)α antibody and CSF-1R small molecule inhibitor BLZ945-loaded hollow mesoporous PB (HMPB) and differentiation inducer all-trans retinoic acid (ATRA) into fibrin for cancer immunotherapy (Fig. 8A). In HMPB/BLZ945/anti-SIRPα@ATRA@fibrin treatment, HMPB exerts CAT-like function, anti-SIRPα antibodies and BLZ945 promote macrophage polarization from M2- to M1-like phenotype by blocking the corresponding CD47-SIRPα and CSF-1/CSF-1R signal axes combined with ATRA-induced differentiation of cancer stem cells, which restores the phagocytosis and killing ability of macrophages to tumor cells, effectively inhibiting postoperative recurrence of hepatocellular carcinoma (HCC).

#### Alleviating tumor hypoxia

In another example, Zhang et al. [194] modified MPBNPs with low molecular weight hyaluronic acid (LMWHA) and loaded with the sono-sensitizer of hematoporphyrin monomethyl ether (HMME) to construct an in situ microenvironmental nano-regulator LMWHA-MPB/HMME for inhibiting 4T1 tumor proliferation and metastasis (Fig. 8B). As a macrophage transducer and oxygen generator, LMWHA-MPB can reshape TAMs phenotype (pro-tumor M2 → anti-tumor M1) in tumor hypoxic areas and continuous supply of H_2_O_2_ by LMWHA to generate sufficient O_2_ to relieve hypoxia through CAT-like catalytic activity of MPBNPs, and facilitate O_2_ self-supplied sonodynamic therapy (SDT). HMME-loaded LMWHA-MPB successfully inhibited the proliferation and metastasis of 4T1 tumors in vivo by improving the tumor microenvironment.

To treat high-risk GBM and simultaneously eliminate CSC and cancer cells, the Liu group constructed a multifunctional nano-platform (G/APH-M) for enhanced radioimmunotherapy [195]. As shown in Fig. 8C, G/APH-M was fabricated by the hollow Prussian blue nanoparticles (HPBNPs) loaded with Gboxin (oxidative phosphorylation inhibitor) and Au-Pt nanozymes and coated with GL261 cell membrane. As a result, G/APH-M were able to achieve BBB-penetrable delivery and GBM-homologous targeting through GL261 cell membranes. In addition, Gboxin and nanozymes released by G/APH-M can kill CSCs by inhibiting mitochondrial oxidative phosphorylation and enhancing radiotherapy by catalyzing O_2_ production, respectively. When G/APH-M combined with immune checkpoint suppression (αPD-L1) induced a strong ICD of GBM and triggered a significant anti-tumor immune response with an 80% survival rate in the orthotopic GBM model even at 60 days after the treatment.

#### Self-supplying oxygen and lactate depletion

High acidity is another characteristic of the tumor microenvironment, which is mainly caused by lactate, a tumor glycolytic metabolite [196]. Recently, studies have shown that lactic acid content in TME ranging from 4 mM to 40 mM leads to immunosuppressive TME and affects the function of immune cells, including damaging tumor cytotoxic T lymphocytes and “feeding” the Tregs [197, 198]. Therefore, the regulation of acidic TME by consuming lactate is a promising cancer immunotherapy. Zhang et al. [199] proposed an acidity modulation combined with programmed death ligand-1 (PD-L1) siRNA (siPD-L1) strategy for synergistically enhancing tumor immunotherapy (Fig. 8D). The lactate oxidase (LOx) was encapsulated into HPBNPs and modified with polyethyleneimine (PEI) and polyethylene glycol (PEG) via sulfur bonds in the outermost layer and loading with siPD-L1 via electrostatic adsorption to obtain HPB-S-PP@LOx/siPD-L1. The co-delivery nano-vector has the characteristics of stable systemic circulation and tumor tissue accumulation and can release LOx and siPD-L1 synchronously in response to high levels of glutathione (GSH) in TME and avoid lysosomal degradation. Moreover, Lactate was first catalyzed by LOX to produce H_2_O_2_, and then further catalyzed by HPB-S-PP to produce O_2_, which in turn promotes the oxidation of lactate in hypoxic tumor tissue. The acidic TME regulation via lactate depletion can transform immunosuppressive TME into immunocompetence TEM, including revitalizing the exhausted CD8 + T cells decreasing the proportion of immunosuppressive Tregs, and synergistically elevating the therapeutic effect of PD1/PD-L1 blockade via siPD-L1.

#### Reprogramming hypoxic and immunosuppressive tumor microenvironment

Jie et al. [200] reported a multimodal therapeutic approach for inhibiting tumor recurrence and metastasis by integrating ICB with anticancer drugs, PTT, and hypoxia relief using PBNPs. To achieve this collaborative approach, they developed a multifunctional nanoplatform called SP94-PB-SF-Cy5.5 NPs (Fig. 8E). This nano-platform contained PBNPs loaded with sorafenib (SF), a chemotherapy drug, conjugated with HCC-targeting peptide SP94 and a near-infrared dye cyanine (Cy) 5.5, which increased the concentration of SP94-PB-SF-Cy5.5 NPs at HCC tumor sites, minimized the side effects of SF, and effectively inhibited tumor local recurrence. Furthermore, this nanoplatform induced ICD to promote DC maturation by alleviating tumor hypoxia; decreasing M2 macrophages, T_reg_ cells, and MDSC; and increasing CTL infiltration, resulting in an immune-promoting TME. When integrated with anti-PD-L1 mAb, the SP94-PB-SF-Cy5.5 NPs inhibited tumor metastasis and recurrence, contributing to the strong abscopal effects and the induction of long-term immunological memory.

To conclude, owing to the enzymatic characteristics of PBNPs that mimic CAT activity to convert H_2_O_2_ to O_2_, the immunosuppressive features of the TME can be modulated using PBNPs to improve the physicochemical properties of the TME including but not limited to hypoxia, acidity. Therefore, PBNPs-based nanoparticles were expected as a TME regulatory tool to improve the therapeutic outcomes of immunotherapies by alleviating TME hypoxia, polarizing TAMs into inflammatory M1 macrophages, decreasing the activity of immunosuppressive T_reg_ cells, and increasing the infiltration of immune cells.

### Prussian blue-based nanoparticles for iron therapy and immunotherapy

#### Ferroptosis-mediated immunotherapy

As a crucial nutrient element, iron is involved in the regulation of several proteins. Maintaining iron homeostasis is important for the function of ferritin, including ATP production, DNA synthesis and repair, and oxygen transport. Furthermore, disruptions in iron metabolism can lead to iron overload or iron deficiency and contribute to tumor growth and progression. Previous studies have reported the importance of proteins and genes associated with iron ion metabolism in the immune system. Notably, iron homeostasis networks are being explored to develop novel tumor immunotherapy strategies [201–203]. Cells mainly export ferrous into the bloodstream via transferrin. Furthermore, the negative feedback of hepcidin regulates iron flux by binding to iron transporters and directly blocking and inducing internalization, ubiquitination, and degradation of the ferroportin (FPN)-hepcidin complex, thereby inhibiting Fe (II) efflux [204]. Fe (II) present in the cytoplasm forms an unstable iron pool. Fe (II) generates ROS after the Fenton reaction, further inducing lipid peroxide accumulation and ferroptosis [205, 206]. The N6-methyladenosine (m^6^A) modification of mRNAs is closely associated with ferroptosis. The activity of enzymes involved in epigenetic modification is largely dependent on Fe(II) activity [207]. For instance, the fat mass and obesity-associated protein was the first identified RNA demethylase to remove methyl from m^6^A in a Fe(II) and α-ketoglutaric acid (α-KG) dependent manner [208]. Hence, intracellular Fe(II) levels can regulate sensitivity toward ferroptosis.

Iron-based nanoparticles can start the Fenton reaction and induce ferroptosis of cancer cells due to their ability to transport exogenous iron to cancer cells [209]. Furthermore, the release of DAMPs during ferroptosis can further activate the immune response, providing a feasible strategy for iron-based nanomaterial-enhanced tumor immunotherapy [210]. As special iron-based nanoparticles, PBNPs can deliver and release iron ions in tumors via the surface modification of tumor-targeting components, which is a promising inducer of ferroptosis. Previous studies have revealed a mutual association between ferroptosis and ICB in tumor cells. For instance, CD8 + T cells activated by ICB promote ferroptosis by inhibiting GPX4. Ferroptosis contributes to ICB by accelerating the rate of tumor infiltration of T lymphocytes.

**Fig. 9:**
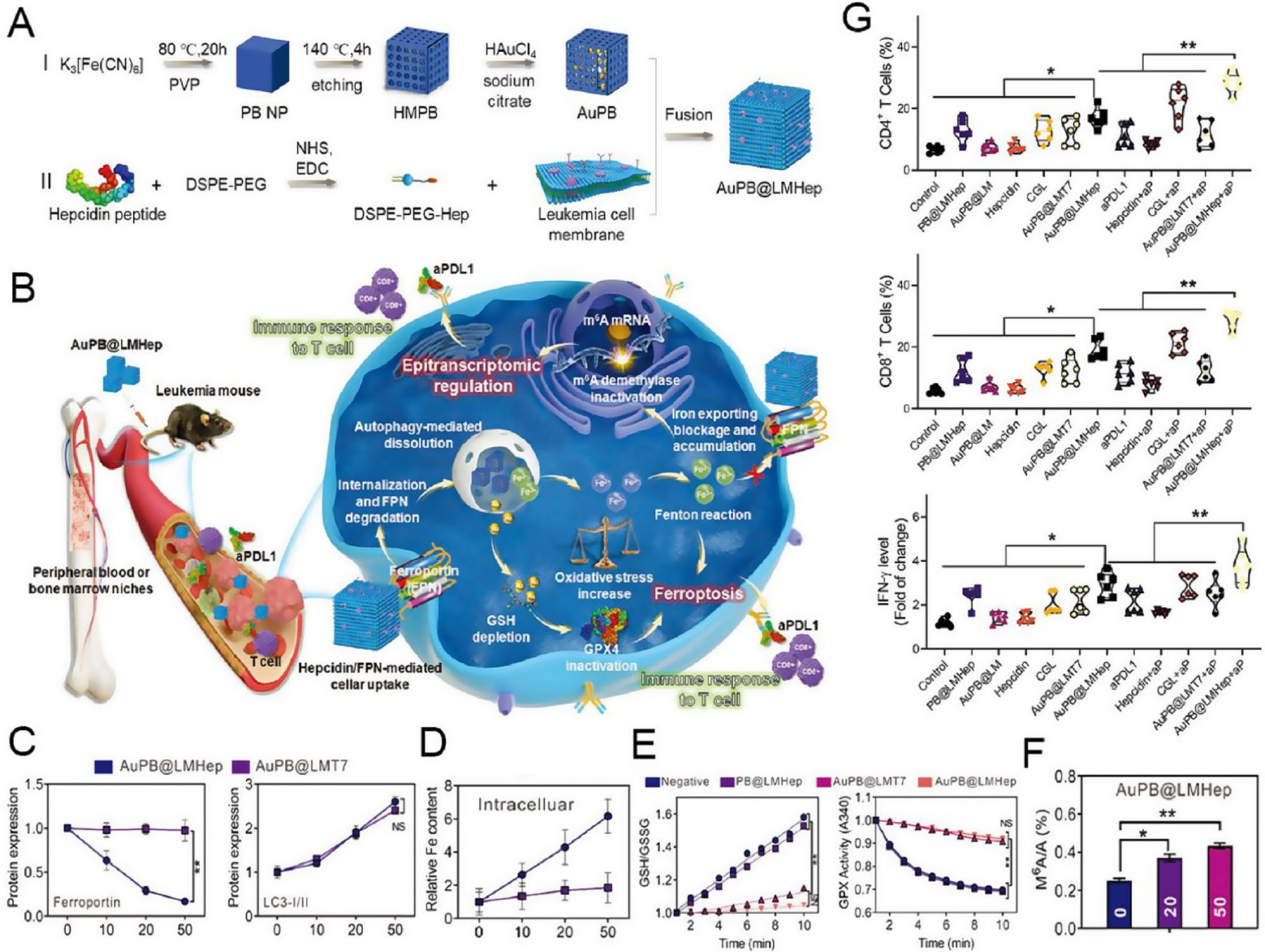
(**A**) Schematic illustration showing the preparation of AuPB@LMHep. (**B**) the hepcidin-based nanocomposites for enhanced cancer immunotherapy by modulating iron export-mediated N6-methyladenosine RNA transcript. (**C**) Protein expression of ferroportin and LC3-I/II in Kasumi-1 cells treated with AuPB@LMHep or AuPB@LMT7 (0 to 50 µg mL^− 1^). (**D**) Intracellular Fe content in AuPB@LMHep or AuPB@LMT7 treated Kasumi-1 cells. (**E**) GSH/GSSH ratio and GPX4 activity in Kasumi-1 cells. (**F**) Dot blots showing m^6^A levels in the Kasumi-1 cells after AuPB@LMT7 treatment. (**G**) Quantification by flow cytometry of ratio of CD8 + T cell and CD4 + T cells populations, as well as Elisa assay of IFN-γ in the different treatment groups of leukemia-bearing mice. Reproduced with permission [211]. Copyright 2021, Wiley-VCH GmbH

Zhang et al. [211] developed an iron-based therapeutic nano-platform (AuPB@LMHep) by integrating hepcidin and leukaemia cell membrane vesicles on hollow mesoporous PB loaded with gold nanorods for improving ICB (Fig. 9A **and B**). Hepcidin-modified AuPB@LMHep significantly downregulated the expression of ferroportin (Fig. 9C), which blocked transferrin-mediated iron exporting and led to intracellular iron accumulation (Fig. 9D), which synergistically triggered ferroptosis with Au-induced GSH depletion and GPX4 inactivation (Fig. 9E). Furthermore, AuPB@LMHep also inactivated the endogenous iron-dependent m^6^A demethylase, increased total m^6^A RNA modification (Fig. 9F), and downregulated genes (FTO and ALKBH5) associated with immune response. AuPB@LMHep + anti-PD-L1 treatment increased the number and infiltration of CD8 + T cells and CD4 + T cells and the levels of secreted IFN-γ (Fig. 9G), resulting in a potent T-cell immune response against leukaemia. Altogether, this multifunctional nanoplatform exhibited a superior anti-tumor effect by synergistic ferrotherapy and ICB.

#### Regulating tumor-associated macrophages polarization by iron ion

Iron homeostasis plays a major role in modulating TAMs polarization through diverse pathways, including intracellular iron levels, iron-mediated ROS, and lysosomal autophagy. Increased iron levels of TAMs promote proinflammatory M1 macrophages while inhibiting anti-inflammatory M2 macrophages. Iron-associated genes are differentially expressed between M1 and M2 macrophages. Furthermore, M2 macrophages release iron into the TME; thus, their iron levels are generally lower than those in M1 macrophages [212, 213]. This finding supports the notion that macrophage polarization influences cancer progression through iron-dependent mechanisms. Besides acting directly on tumor cells, PBNPs also supply a significant source of iron to macrophages, enabling them to reverse TAMs immunosuppression by regulating macrophage polarization.

**Fig. 10:**
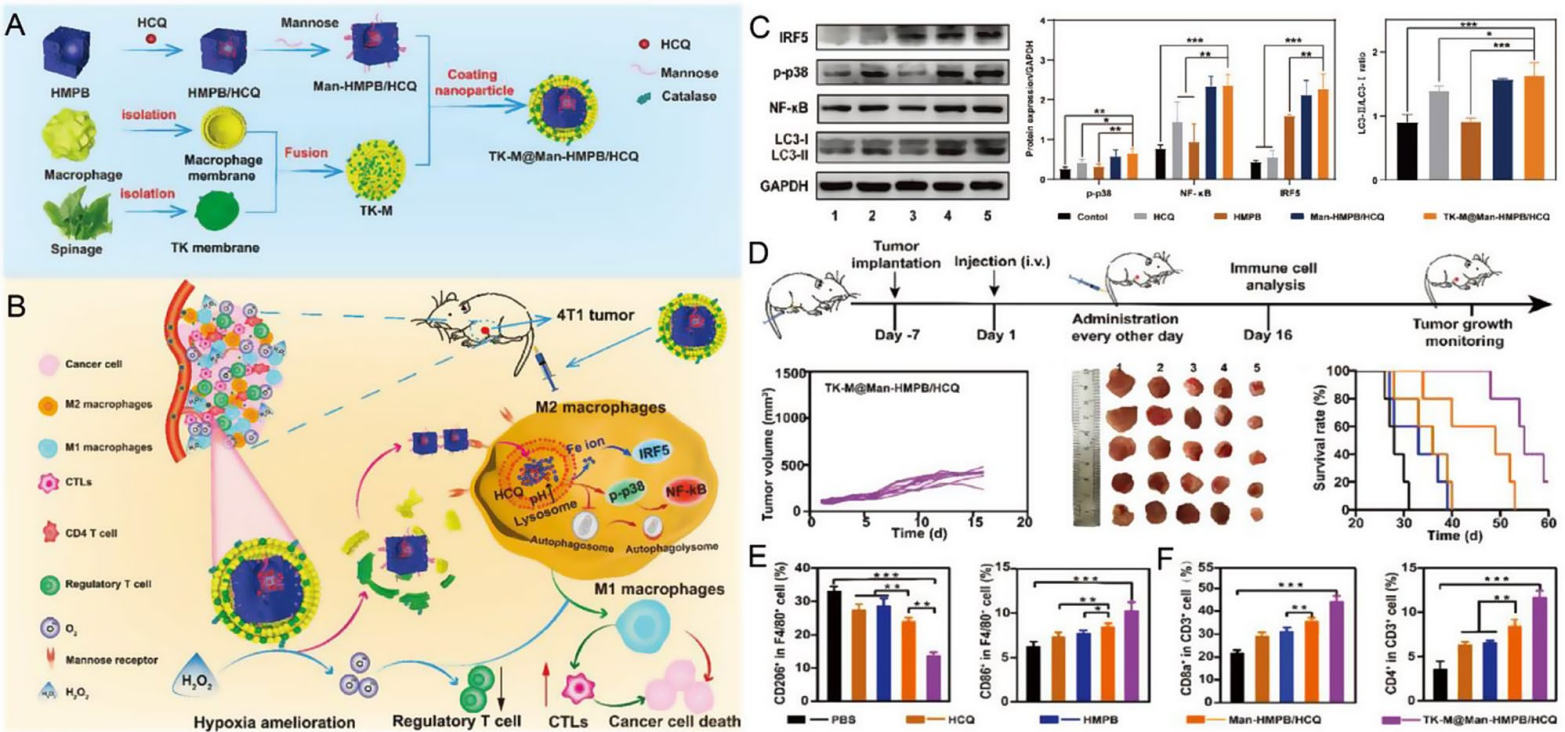
(**A**) Schematic diagram of the preparation of Man HMPB/HCQ coated with TK-M hybrid membrane. (**B**) TK-M@Man-HMPB/HCQ potentiates cancer immunotherapy via mitigating hypoxia, reversing the TAMs phenotypes, and facilitating cytotoxic T lymphocyte infiltration. (**C**) Western blot and corresponding semi-quantitative analysis of M2 macrophages (RAW264.7 treated with IL-4 for 24 h) after different treatments. (**D**) In vivo antitumor efficacy investigation. (**E**) Relative quantification of M2-like macrophages (CD206+) and M1-like macrophages (CD86+) gating on F4/80 + cells (*n* = 5, mean ± SD). (**F**) Relative quantification of CD8 + and CD4 + T cells gating on CD3 + T cells (*n* = 5, mean ± SD). *p < 0.05, **p < 0.01, ***p < 0.001, by analysis of ANOVA with Tukey’s post-hoc test. Reproduced with permission [22]. Copyright 2022, Wiley-VCH GmbH

Hou et al. [22] synthesized a mannose-modified HMPB nanosystem that encapsulates hydroxychloroquine (HCQ). This nanosystem was coated with a hybrid membrane of macrophages and thylakoids (TK) (TK-M@Man-HMPB/HCQ) to polarize TAMs and reduce hypoxia, enhancing the efficacy of cancer immunotherapy (Fig. 10A **and B**). The TK-M@Man-HMPB/HCQ nanosystem is capable of being internalized by RAW264.7 cells through the targeting of the mannose receptor. Additionally, iron ions and HCQ are released from the nanosystem. This synergically induces TAMs polarization via the activation of the IRF5 pathway and inhibition of autophagy of M2 macrophages, respectively (Fig. 10C). Furthermore, TK-M@Man-HMPB/HCQ notably facilitates cancer cell death (Fig. 10D) and promotes the infiltration of CTLs while inhibiting the growth of regulatory T cells.

In general, PBNPs tend to induce the anti-inflammatory M2 phenotype. However, the polarization of macrophages induced by iron-based nanoparticles is not only affected by the inherent physicochemical properties of nanoparticles (including composition, size, surface, etc.), but also regulated by various external factors such as magnetic field, laser irradiation and temperature [214]. For example, the smaller Fe_3_O_4_ NPs 4 nm triggers M1 polarization more efficiently than the 14 nm Fe_3_O_4_ NPs [215]. PBNPs coated with LMWHA promoted phenotypic reversal of M2 to M1 in RAW264.7 cells [216]. More importantly, the regulation of macrophage polarization by iron-based nanoparticles involves multiple mechanisms, including membrane receptor interference, transcriptional regulation, ROS rebalancing, lysosomal autophagy pathway, and iron ion release. Although the above study shows a special case of PBNPs triggering M1 polarization, it is the basic problem to be considered in future research: how can iron-based nanoparticles be rationally designed to achieve precise modulation and control of macrophage polarization?

### Prussian blue-based nanoparticles for multimodal synergistic therapy and immunotherapy

Multimodal therapy involving immunotherapy in conjunction with other therapies can be accomplished by encasing various therapeutic compounds in PBNPs, potentially improving the anti-cancer and reducing side effects. We have collected the following research strategies: (1) a combination of PTT/CDT for enhancing immunotherapy via stimulating antigen presentation and remodelling the tumor microenvironment, (2) targeting chemo-photothermal therapy to enhance anti-PD-L1 efficiency via robustly inducing pyroptosis, (3) integration of theranostic performance (NIR-II fluorescence and PDT/PTT) of aggregation-induced emission luminogen (AIEgen) with nano-catalytic property of PBNPs for robust cancer immunotherapy, (4) strategy involving photothermal ablation and hypoxic reversal to potentiate the STING-dependent innate antitumor immunity.

**Fig. 11:**
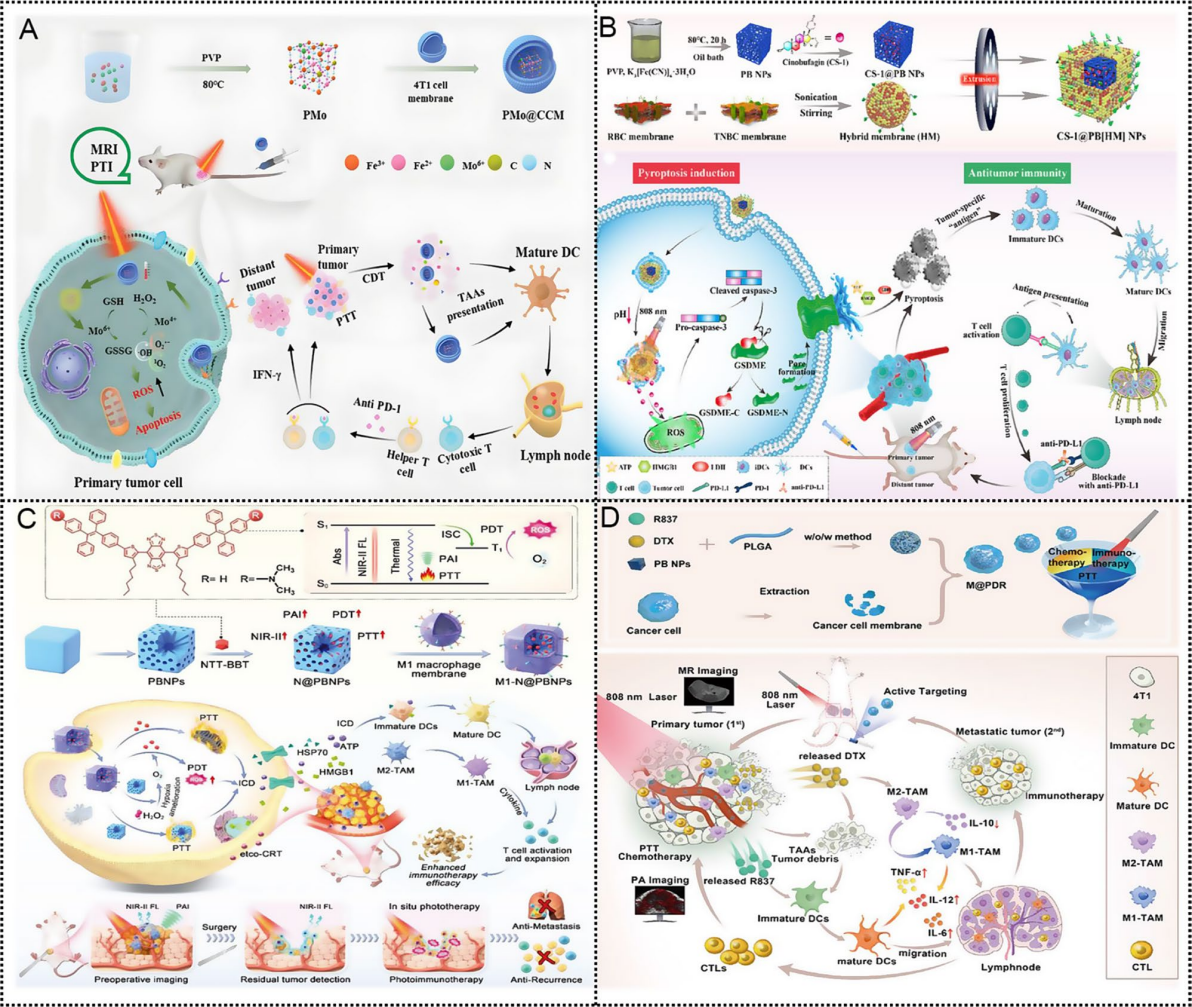
(**A**) Schematic illustration of the synthesis of PMo@CCM and the pathway of enhanced immunotherapy via the combined PTT/CDT treatment and improver presentation of tumor-associated antigens (TAAs) [217]. Copyright 2024, Wiley-VCH GmbH. (**B**) Schematic illustration of the CS-1@PB[HM] NPs for cancer comprehensive therapy by inducing pyroptosis [218]. Copyright 2023, Elsevier Ltd. (**C**) Schematic illustration showing the integration of NIR AIEgen with mesoporous PB nanocatalyzer to boost the theranostic performance for NIR-II fluorescence and PA imaging-guided robust cancer immunotherapy [219]. Copyright 2024 Wiley-VCH GmbH. (**D**) Schematic diagram of the homologous targeted tumor cocktail therapy based on M@P-PDR “Nano-targeted cells” [228]. Copyright 2021, BioMed Central

PTT and CDT induce tumor cell death to release TAA due to their ability to induce local hyperthermia and production of reactive oxygen species (ROS), respectively, and these antigens are then captured by dendritic cells (DC) and subsequently catalyze T-cell-mediated anticancer immune activation. PTT and CDT synergies not only ensure effective and direct tumor ablation but also have great potential to enhance the immune response. Wang et al. [217] developed a molybdenum-doped Prussian blue (PMo) nanoparticles that further encapsulated 4T1 cancer cell membranes to form PMo@CCM (Fig. 11A). The incorporation of molybdenum not only significantly improves the efficiency of PTT, but also enhances CDT by producing ROS and consuming GSH. The combination of PTT and CDT effectively induced ICD and promoted antigen presentation. CCM coatings provide a rich combination of TAA with PTT and CDT to significantly stimulate DC maturation, activate T cells, and enhance the overall efficacy of immunotherapy. In addition, further administration of programmed cell death protein 1 antibodies (anti-PD-1) enhanced their ability to recognize and eliminate tumor cells by targeting the PD-1 protein expressed in T cells. The synergistic interaction between PTT, CDT, and immunotherapy not only showed significant efficacy against the primary tumor but also effectively inhibited the growth of distant tumors.

Liu et al. [218] designed a novel tumor-targeting nanomedical drug (CS-1@PB[HM] NPs) using PBNPs loaded with the antitumor drug CS-1 and disguised with hybrid membranes (erythrocyte and TNBC membranes). As shown in Fig. 11B, this nanomedicine CS-1@PB[HM] NPs not only exhibits deep penetration in tumor tissues, but also releases CS-1 in a photothermally controlled manner, inducing strong pyrolysis and GSDME-dependent tumor cell pyrolysis, promoting the release of tumor antigens and DAMPs, and finally inducing DCs maturation, which effectively stimulates the adaptive anti-tumor efficacy and improves the anti-PD-L1 efficacy.

Photodynamic therapy (PDT) is another type of phototherapy that can produce ROS, but different from CDT, PDT is highly dependent on oxygen. However, hypoxic TME inevitably undermines the efficacy of PDT. In addition, hypoxic TME properties promote immunosuppression through various mechanisms, especially tumor-associated macrophage (TAMs) recruitment. Therefore, strategies that combine photoimmunotherapy with oxygen regulation are expected to address the challenges faced by photoimmunotherapy. Shen’s group reported a high-performance phototherapy diagnostic platform with fine engineering characteristics by incorporating AIEgens into PBNPs [219]. As shown in Fig. 11C, PBNPs effectively limits the molecular motion of AIEgen, thereby amplifying fluorescence brightness and improving PDT properties. On the one hand, PBNPs promotes the PDT effect of AIEgen by producing oxygen in situ, on the other hand, it enhances the photoacoustic imaging (PAI) and PTT effect of AIEgen by NIR absorption capacity.

Each PAMP is recognized by a different TLR, and the natural ligand of TLR7 is a single-stranded RNA rich in guanyl and uridine [220]. The small molecule immunoadjuvant Imiquimod (R837) [221], a member of the imidazolines family, is the first U.S. FDA-approved TLR7 agonist for treating genital warts [222], actinic keratosis [223], and superficial basal cell carcinoma in patients (age ≥ 12 years) [224]. TLR7 is mainly located in the endosomal compartment of DC and B cells and exists as a monomer. After ligand binding, dimerization triggers the recruitment of MyD88, the translocation of downstream IFN regulatory factor (IRF)7, the activation of nuclear factor-κb, and finally the production of proinflammatory cytokines, IFN-I, and chemokines [225]. Imiquimod can induce notable DC maturation after binding to TLR7 and promote DC migration from skin to draining lymph nodes, enhancing tumor-specific cytotoxic CD8 + T cells and macrophage activation [226]. Despite presenting great potential in local and systemic cancer immunotherapy, the application of imiquimod as a monotherapy is still limited by its poor water solubility, serious adverse reactions when administered in a large dosage, and possible immune tolerance to TLR7 [227]. A promising strategy may be the synergistic therapy of imiquimod plus PBNPs-PTT. Ran et al. [228] reported that “Nano-targeted cells” (M@P-PDR) nanospheres, which were fabricated with PB NP-encapsulated and docetaxel (DTX)/R837-loaded poly (lactic-co-glycolic acid) (PLGA) nanospheres coated with cell membranes for cocktail therapy that integrates several therapeutic modalities, including chemotherapy, immunotherapy, and PTT (Fig. 11D). M@P-PDR effectively promoted DC maturation and migration to lymph nodes by releasing immunoadjuvant R837, along with increasing proinflammatory cytokine (such as tumor necrosis factor α, interleukin IL-6, and IL-12) production by releasing chemotherapeutic drugs DTX to polarize M2-phenotype TAMs to M1-phenotype, enhancing CTL infiltration. Cocktail therapy based on M@P-PDR “Nano-targeted cells” compensated for the deficiencies of each monotherapy and produced a superimposed effect on tumor regression and metastasis/recurrence.

In short, multimodal combination therapy aims to target tumors more comprehensively through synergies and has become a trend to improve the clinical efficacy of cancer. These combination therapies involve not only the combination of different immunotherapies, but also the combination of them with cancer therapies that target the tumor microenvironment (TME), traditional chemical/radiation therapy, and emerging phototherapy. PB-based nanoparticles have rich physical and chemical properties and biological functions, including as drug delivery carriers for integrating multi-component drugs to implement multi-synergistic therapy in a single nano platform and as functional nanomedicine for PTT, enzyme-catalyzed therapy and ferroptosis therapy one or more and further combined therapy with other drugs. However, the development of scientific and rational combination therapy strategies relies on an in-depth analysis of tumor biology and immune status and requires a deep understanding of the mechanisms of action of different drug combinations.

## Conclusions and outlook

**Fig. 12:**
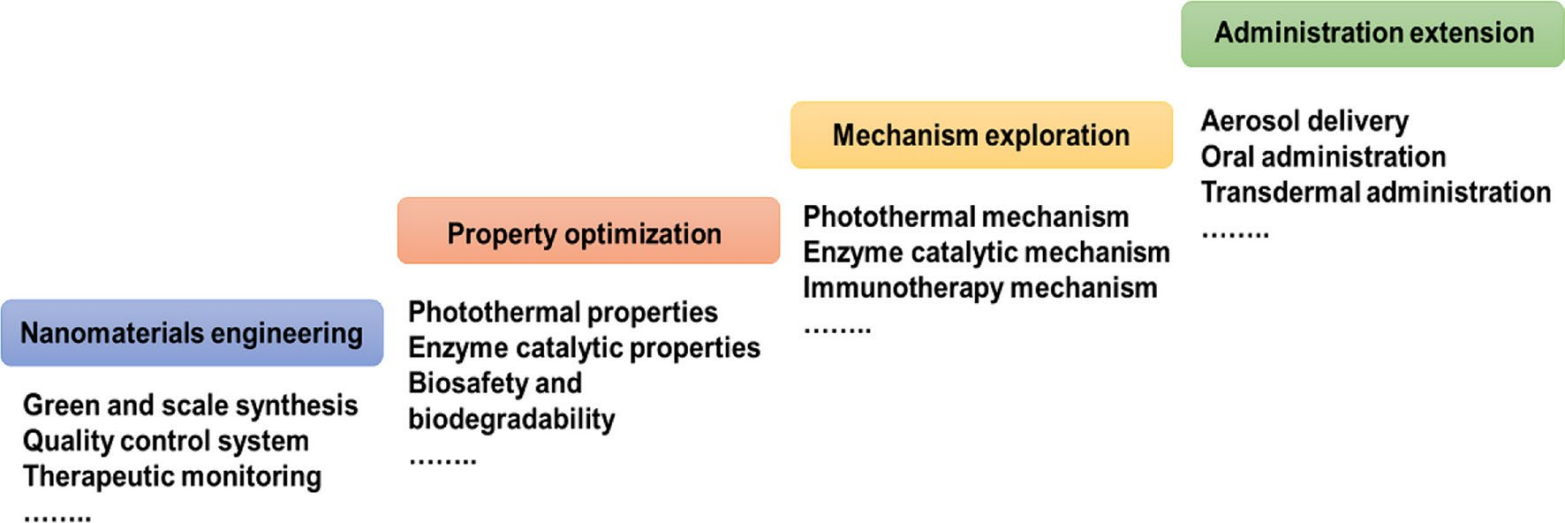
The current challenges and future development of PBNPs in tumor immunotherapy

The advantageous features of the iron active centre, MOF structure, straightforward synthesis, and facile modification render PB-based nanoparticles versatile tools that can substantially trigger anti-tumor immune responses through various mechanisms, providing clear benefits over other nanoparticles in cancer immunotherapy. In this review, the composition, structure, and physicochemical properties of PBNPs are initially presented, establishing the foundation for their versatility in nanomedicine as nanocarriers, PAT, nanozymes, and iron donors. Crucially, the recent advancements in PBNPs-based PTT and immunotherapy have been systematically reviewed. This includes PBNPs-based PTT for initiating ICD, as well as the synergistic therapy of PBNPs-PTT with immune adjuvants, ICB, or ACT. Additionally, we discuss How PBNPs improve immunotherapy by modulating TME and by iron therapy, respectively. PBNPs with CAT enzyme-like activity can regulate hypoxic and acidic TME by catalyzing H_2_O_2_ in TME to generate O_2_, making TME less immunosuppressive. PBNPs-based iron therapy can improve immunotherapy efficacy by inducing tumor cell ferroptosis and regulating TAMs polarization by releasing ions ion. To overcome the limitations of monotherapy and minimize the dose and side effects of therapeutic agents, PBNPs-based multi-modal synergistic treatment regimens are being actively developed. Finally, we described the current challenges and future developments of PBNPs in cancer immunotherapy (Fig. 12).

1) The engineering design strategy of PBNPs is the preliminary stage to achieve excellent therapeutic effects. Therefore, it is crucial to develop green and sustainable synthesis methods, simplify synthesis steps, and establish quality control systems for the industrialization and clinical translation of PB-based nanoparticles to reduce synthesis costs. Attention should be directed toward designing an integrated diagnosis and treatment nano-platform for real-time monitoring of therapeutic effects in the immune engineering of PBNPs.

2) It is crucial to optimize the physicochemical properties of PBNPs, which influence the pharmacological parameters of PB-based NPs, including blood circulation, tissue penetration, cell uptake, intracellular transport, controlled release, half-life and metabolism clearance. Therefore, the study of how PB-based NPs interact with biological systems shall be carried out. Although tumor-targeted PBNPs have shown effective tumor accumulation, they are only able to absorb light sources in the first NIR window (NIR-I) with limited tissue penetration depth, resulting in significantly reduced PTT efficacy. Therefore, PB-based nanoparticles with NIR II absorption capacity need to be developed to improve the photothermal efficacy. Besides, the catalytic behavior of PB-based nanozymes is strictly regulated by size and crystallinity. For example, Luo et al. observed that ultra-small Prussian Blue nanozymes (USPBNZ) demonstrated markedly enhanced ROS clearance activity compared to larger-sized PBNZ [229]. Similarly, Zhang et al. found that PBNPs with low crystallinity and small size showed significantly stronger POD-like and CAT-like activities than PBNPs with high crystallinity and amorphous structure [230]. The biosafety and biocompatibility of PBNPs are the primary challenges for its clinical translation, and the FDA has approved PB for oral therapeutic antidotes. However, PBNPs used for tumor therapy are usually administered by intravenous (i.v.) or intratumoral injection, which differs from the pharmacokinetic (PK) characteristics of oral administration and is more likely to induce toxicity risks. Wang et al. [231] comprehensively investigated the fate and risks of PBNPs after intravenous administration using a mouse model and a comprehensive approach of pharmacokinetics, toxicology, proteomics, and metabolomics, and the results showed that high-dose administration of PBNPs (20 mg/kg) may cause potential risks to the liver and lungs of mice, which will provide detailed reference and guidance for further clinical application of PBNPs in the future. Therefore, the biosafety and biocompatibility of PB-based functional nanoparticles for cancer immunotherapy need to be comprehensively evaluated. In addition, currently reported PB-based nanoparticles are simple animal models (mouse) rather than animal models that can accurately simulate human tumor conditions, resulting in the application of PBNPs is still stuck in the preclinical research stage. Due to the high mortality of tumor metastasis, it is more meaningful to evaluate the efficacy of PBNPs in both in-situ and metastatic tumor models.

3) It is necessary to systematically elucidate the photothermal mechanism, catalytic mechanism, and detailed immunotherapy mechanism underlying PB-based nanoparticles, which can provide a novel direction for the design and establishment of multifunctional PB-based nanoparticles. Zhang et al. [232] revealed that PB nanozymes (PBNZ) follow a dual-path electron transfer mechanism during POD and Cat-like catalysis, which has the advantage of long service life. Although PB-based nanoparticles have been widely used in single or combined immunotherapy, our understanding of the immune mechanisms involved remains insufficient, which is critical for the advancement of immunotherapy.

4) The most suitable drug delivery manner for PB-based nanomaterial should be investigated in clinical applications. Although PBNPs via intravenous injections or intratumoral injection have shown ideal anti-tumor effects in preclinical studies, the optimal delivery routes for different cancer types are not known. For digestive system-related cancers, such as gastric cancer and colorectal cancer, oral administration is considered the optimal route. Aerosol administration can be the most effective route for respiratory system-related cancers, such as lung cancer. Furthermore, for skin-related cancers, such as melanoma, transdermal administration may be the optimal way. Hence, it is crucial to study the manner of drug delivery of PB-based nanoparticles in the future, laying the foundation for improving their bioavailability.

This review provides an in-depth understanding of PBNPs and their application in cancer immunotherapy. We aim to make a way for the bench-to-bedside translation of PBNPs in the future. Urgent efforts and multidisciplinary cooperation are required to allow the rapid clinical translation of the PBNP-based nano-immunotherapy platforms.

## Data Availability

No datasets were generated or analysed during the current study.
